# Signature of mid‐Pleistocene lineages in the European silver fir (*Abies alba* Mill.) at its geographic distribution margin

**DOI:** 10.1002/ece3.7886

**Published:** 2021-07-21

**Authors:** Caroline Scotti‐Saintagne, Thomas Boivin, Marie Suez, Brigitte Musch, Ivan Scotti, Bruno Fady

**Affiliations:** ^1^ INRAE Ecologie des Forêts Méditerranéennes (URFM) Avignon France; ^2^ ONF UMR 0588 BioForA Orléans France

**Keywords:** admixture, conservation, demography, French Pyrenees, genetic diversity, keystone species, Phylogeography, Quaternary climate, range‐edge

## Abstract

In a conservation and sustainable management perspective, we identify the ecological, climatic, and demographic factors responsible for the genetic diversity patterns of the European silver fir (*Abies alba* Mill.) at its southwestern range margin (Pyrenees Mountains, France, Europe). We sampled 45 populations throughout the French Pyrenees and eight neighboring reference populations in the Massif Central, Alps, and Corsica. We genotyped 1,620 individuals at three chloroplast and ten nuclear microsatellite loci. We analyzed within‐ and among‐population genetic diversity using phylogeographic reconstructions, tests of isolation‐by‐distance, Bayesian population structure inference, modeling of demographic scenarios, and regression analyses of genetic variables with current and past environmental variables. Genetic diversity decreased from east to west suggesting isolation‐by‐distance from the Alps to the Pyrenees and from the Eastern to the Western Pyrenees. We identified two Pyrenean lineages that diverged from a third Alpine–Corsica–Massif Central lineage 0.8 to 1.1 M years ago and subsequently formed a secondary contact zone in the Central Pyrenees. Population sizes underwent contrasted changes, with a contraction in the west and an expansion in the east. Glacial climate affected the genetic composition of the populations, with the western genetic cluster only observed in locations corresponding to the coldest past climate and highest elevations. The eastern cluster was observed over a larger range of temperatures and elevations. All demographic events shaping the current spatial structure of genetic diversity took place during the Mid‐Pleistocene Transition, long before the onset of the Holocene. The Western Pyrenees lineage may require additional conservation efforts, whereas the eastern lineage is well protected in in situ gene conservation units. Due to past climate oscillations and the likely emergence of independent refugia, east–west oriented mountain ranges may be important reservoir of genetic diversity in a context of past and ongoing climate change in Europe.

## Graphical abstract

Genetic diversity of the silver fir, *Abies alba* Mill., decreases from east to west in western Europe, suggesting isolation‐by‐distance from the Alps to the Pyrenees and from the Eastern to the Western Pyrenees. The two Western and Eastern north‐Pyrenean lineages identified diverged long ago, during the Mid‐Pleistocene Transition, while population size fluctuation occurred later, possibly during the Late Glacial Maximum, with a contraction in the west and expansion in the east. East‐west oriented mountains such as the Pyrenees are important reservoirs of genetic diversity in a context of climate change, making likely the emergence of independent refugia, lineages, and possibly local adaptation, needing conservation measures.

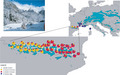

## 1 INTRODUCTION

As the world's biodiversity continues to decline (Secretariat of the Convention on Biological Diversity, 2020), the Post‐2020 Biodiversity Framework of the Convention on Biological Diversity (CBD) and the EU Biodiversity Strategy for 2030 have called for transformative changes that will halt biodiversity decline and maintain ecosystem, species, and genetic diversity for the benefit of people (Hoban et al., 2020).

Among these transformative changes is the need to better consider and protect genetic diversity. It is a key component of resilience and adaptability in its own right and it can be harnessed for the benefit of people using nature‐based innovative management suitable at times of climate uncertainty (Fady et al., 2016; Hoban et al., 2020). Knowledge of how genetic diversity is structured is important for delineating conservation strategies and identifying areas where conservation efforts should be focused (Moritz & Faith, 1998). Populations with the highest genetic diversity are likely to display increased resilience and those harboring different ancestral alleles in past refuge zones are likely adapted to different environments, a crucial information for prioritizing population conservation (Fady et al., 2016; Fernandez‐Fournier et al., 2021; Hampe & Petit, 2005).

A significant number of phylogeographic studies have demonstrated the role of Holocene and the Last Glacial Maximum in the distribution of species, populations, and their genetic diversity in the world's temperate regions, emphasizing climate change and dispersal as the main drivers of observed and modeled patterns. Current species geographic distribution in the Northern Hemisphere is often the result of a rapid south‐to‐north colonization during the warm Holocene period that followed the Last Glacial Maximum (Hewitt, 1999). The consequence for European plants is a strong latitudinal gradient of genetic diversity, with often higher values in the south than in the north (Petit et al., 2005), although reverse clines have been observed, for example, for Artic‐Alpine plants which also demonstrate lower diversity margin effects (Hirao et al., 2017). In the Mediterranean basin, where major glacial refugia were located, genetic diversity shows an additional, longitudinal gradient, particularly in trees and insects, with higher diversity in the east than in the west (Fady & Conord, 2010), interpreted as resulting from both east‐west Holocene migrations and differential local drift during the Last Glacial Maximum (see e.g., the widespread forest tree *Pinus nigra*, Scotti‐Saintagne et al., 2019).

Additionally, less widespread evidence indicates that earlier Pleistocene climate and geological events have also shaped current within‐species genetic diversity (Riddle, 2016). While Holocene warming has undoubtedly affected genetic diversity patterns in many species, earlier events of colonization and successive vicariance at different times (Kropf et al., 2006; Schmitt et al., 2006) may also have had a strong impact on current genetic diversity (Avise, 2000). This may be particularly true for long‐lived, sessile organisms such as forest trees. In Europe, forest tree taxa have repeatedly shifted their distributions and disappeared locally throughout the Pleistocene (Magri et al., 2017), resulting in earlier and deeper genetic differentiation patterns than those determined by the last glacial cycle, such as with *Cupressus sempervirens* (Bagnoli et al., 2009) or *Quercus cerris* (Bagnoli et al., 2016) for example.

Past climate changes have had a significant impact on the genetic structure of forest tree populations, and current human‐induced, strong climatic changes can be expected to modify it again. Focusing on populations at the warm margin of their range is of particular relevance as they represent limits to species distributions, which habitats may be strongly altered by climatic change (Crawford, 2008). Silver fir (*Abies alba* Mill.) is a target European conifer species for conservation programs. It is an economically important species as well as a keystone species which maintains high biodiversity forest ecosystems (Mauri et al., 2016). While not threatened *per se*, widespread mortality has been observed recently, particularly at habitat and geographic margins (Cailleret et al., 2014; Camarero et al., 2011).

The distribution range of silver fir is wide but patchy, from southern Poland to northern Spain (Wolf, 2003). The genetic diversity of *Abies alba* is high, indicating that despite geographic patchiness, gene flow is high, but generally declining from east to west (Fady & Conord, 2010; Mosca et al., 2012). At a broad scale, genetic diversity is structured into several lineages resulting from past climate changes. The Italian Peninsula populations were found to have diverged from Balkan populations during the middle Pleistocene (Belletti et al., 2017; Piotti et al., 2017), while the Eastern and Western Alps lineages derived from Balkan and Italian refugia are believed to have merged during the Holocene (Cheddadi et al., 2014; Liepelt et al., 2009). At their south‐westernmost Alpine margin, silver fir populations belong to two different genetic groups, the Southern Alps cluster which also includes the Massif Central populations and the Pyrenees cluster (Liepelt et al., 2009; Sancho‐Knapik et al., 2014). Evidence of local adaptation to drought and frost has been reported in both the Alps and the Pyrenees (Csilléry et al., 2020; Matías et al., 2016; Roschanski et al., 2016).

Compared to the Alps, where the spatial distribution of genetic diversity has been intensively studied, little information is available for the Pyrenees. The geographic distribution of silver fir there is fragmented, as a result of both past climate changes and human impact during the Antiquity (grazing or logging for industrial activity) and until the industrial revolution (Ejarque et al., 2009; Galop & Jalut, 1994; Pérez‐Sanz et al., 2013). This fragmentation has led to the isolation of old‐growth (sensu FAO, 2015) native forests, many of them in marginal habitats where biodiversity conservation issues are most pressing in Europe (Sabatini et al., 2018).

The Pyrenees are the south‐westernmost large alpine‐type ecosystem in Europe making it a hotspot of diversity and endemism (Nagy et al., 2003; Ozenda, 1985; Villar & Dendaletche, 1994) as well as a glacial refugium for many mountain species (Duriez et al., 2007; Horn et al., 2009; Taberlet et al., 1998). The Pyrenees’ longitudinal structure, stretching 450 km from west to east between the Atlantic Ocean and the Mediterranean Sea, confers them unique ecological characteristics. Along the Pyrenees, the landscape changes from oceanic and lower elevation in the west to Mediterranean and variable elevation and geomorphology toward the east (Ninot et al., 2007). These longitudinally contrasted environments have led to a west‐east genetic divergence attributed to secondary refugia in many animal species, such as the mountain ringlet butterfly *Erebia epiphoron* (Schmitt et al., 2006), the ground‐dwelling spider *Harpactocrates ravastellus* (Bidegaray‐Batista et al., 2016), the pine processionary moth *Thaumetopoea pityocampa* (Rousselet et al., 2010), and plant species such as snapdragons *Antirrhinum* sp. (Liberal et al., 2014), *Rhododendron ferrugineum* (Charrier et al., 2014), the clover *Trifolium alpinum* (Lauga et al., 2009), the European oaks (Petit et al., 2002), the European beech *Fagus sylvatica* (Magri et al., 2006), and silver fir (Matías et al., 2016; Sancho‐Knapik et al., 2014). In these animal and plant phylogeography studies, identified lineages are always shared between France and Spain at any given longitude, indicating that the east‐west ridge of high peaks in the Central Pyrenees did not act as a barrier to migration.

Here, we analyze the phylogeography of silver fir with a Northern Pyrenean focus, through detailed landscape‐scale sampling and using neighboring silver fir populations (located in the higher latitude French mountain ranges of the Massif Central, the southern Alps, and Corsica) as external references. We addressed the following questions:

1. What is the evolutionary origin of the Pyrenean lineages? How and when did they diverge from each other and from Alpine lineages?
2. What are the locations of the geographical boundaries between genetic lineages along the Pyrenees, and is there admixture between them?
3. Which demographic events related to past climate oscillations have left the most significant imprint on genetic diversity and can this information be used for furthering *in‐situ* gene conservation efforts in France?

For this, we sampled the Northern Pyrenees only, in France, thus assuming that, as for all current mid‐latitude Northern Hemisphere conifers, *Abies alba* ancestors expanded southward across Europe from circumboreal high latitudes and diversified during the general cooling of the late Eocene and Neogene period, starting some 25 million years ago and culminating during the Pleistocene (Barnosky, 1987; Rundel, 2019; Xiang et al., 2015). We expect that our results will provide a phylogeographic picture valid for the entire Pyrenees (both France and Spain) as range‐wide evidence from other species does not demonstrate the existence of separate lineages on each side of the central high peak ridge of the Pyrenees.

## 2 METHODS

### 2.1 Population sampling and DNA extraction

Sampling consisted of an average of 30 trees per population from a total of 52 populations (Table S1.1 in Appendix S1). Forty‐four populations were sampled spanning a 400 km east/west axis in the French Pyrenees between the Mediterranean and the Atlantic coasts. The Western Pyrenees populations (PYR_W) were sampled in the area between the Bay of Biscay and the Pic d'Anie, where elevation does not exceed 2,000 meters above sea level. The Central Pyrenees populations (PYR_C) were sampled between the Pic d'Anie and the Puymorens pass, in the highest part of the Pyrenees with peaks higher than 3,000 m. The Eastern Pyrenees populations (PYR_E) were sampled in the area east of the Puymorens pass down to the Mediterranean coast; their highest elevation (Puigmal peak, 2,910 m) is intermediate between that of the Central and Atlantic Pyrenees. To compare the genetic diversity observed in the Pyrenees with surrounding mountain ranges, we also sampled four populations in the Massif Central: three in the Western Alps and one in Corsica (collectively defined as “Alpine populations” as they belong to the same lineage, Liepelt et al., 2009).

We sampled wild populations large enough (1.5–3 ha) to allow sampling trees at least 20 meters apart from each other. Sampling consisted of three cambium disks per tree, frozen in liquid nitrogen in portable devices during collection. DNA extraction followed the protocol of Demesure et al. (1995).

### 2.2 Microsatellite genotyping

Among the 52 sampled populations, 50 were analyzed at both chloroplast (cpSSR) and nuclear (nuSSR) microsatellites and two from Western Alps at only one type of marker (VES at nuSSR and LURE at cpSSR).

Three cpSSRs, Pt30141 (Liepelt et al., 2001), Pt15169, and Pt71936 (Vendramin & Ziegenhagen, 1997), were amplified by polymerase chain reaction (PCR) using the protocol of Vendramin and Ziegenhagen (1997). cpSSR pt15169 displays an 18 base pair insertion–deletion (indel), whereas the other two display mononucleotide indel patterns. Genotypes were scored using an ABI PRISM 3100 automatic sequencer (Applied Biosystems) at ONF in Orléans (France), and allele size was assigned using GeneMapper v3.7. In addition, we used ten nuclear microsatellites (nuSSR): six from Cremer et al. (2006) (SF1, SFb4, SFb5, SF50, SF78, and SF333) and four from Hansen et al. (2005) developed for A*bies nordmanniana* and transferable to *A. alba* (NFF3, NFH15, NFF7, NFH3). One nuSSR (SF1) displays a trinucleotide pattern, whereas all the others are dinucleotides. The ten nuSSR were genotyped in two multiplexes using the Multiplex PCR Kit (QIAGEN). The final PCR volume was optimized to 10 μl, and the PCR mix for both multiplexes was 5 μl of the QIAGEN Multiplex PCR Master Mix, 2 μl of DNA (10 ng/μl), and 0.2 μM primers. For both multiplexes, the PCR thermal profile was the following: an initial step at 95°C for 15 min, followed by 35 cycles at 95°C for 30 s, 57°C for 90 s, and 72°C for 90 s, with a final 30‐min extension step at 60°C. PCR products were scored at INRAE in Avignon (France) using an AB 3730 XL automatic sequencer (Applied Biosystems) with LIZ‐600 as internal size standard.

### 2.3 Statistical analysis

Statistical computing of data was made using R version 3.6.3 (R Core Team, 2020) on RStudio (RStudio Team, 2019).

#### 2.3.1 Genetic diversity

Estimation of the null allele frequencies at each locus was performed in each population using the maximum likelihood approach and the EM algorithm of Dempster et al. (1977) available in the Genepop v4.6.9 R package (Rousset, 2008). The proportion of alleles that are unique (in only one individual in the population) *A*_u_ was computed for each population using the StrataG v2.4.905 R package (Archer et al., 2017). The number of alleles *N*_A_, the number of effective alleles *N*_Ae_ (Nielsen et al., 2003), the genetic diversity *H*_e_ corrected for sample size (Nei, 1978), and the inbreeding coefficient *F*_i_ (for nuSSR) were estimated using Spagedi1.4b (Hardy & Vekemans, 2002). Allelic richness and private allelic richness were estimated using rarefaction methods, as implemented in ADZE (Szpiech et al., 2008), to take into account heterogeneity in sample sizes. The number of rarefied gametes was 50 for nuSSRs and 22 for cpSSRs. The distribution of genetic diversity among the sampled populations was explored using a principal correspondence analysis (PCA) performed on the allelic frequencies at the individual level using the R package Adegenet (Jombart, 2008).

#### 2.3.2 Isolation by distance and phylogeographic signals

Pairwise differentiation between populations and between mountain ranges was computed using GENEPOP 4.7.5 (Rousset, 2008). A phylogenetic tree using the distance matrix of *F*_ST_ between populations was built with Poptree (Takezaki et al., 2010) using the neighbor‐joining method (Saitou & Nei, 1987). We used the genotypic data of four Mediterranean firs, *A. pinsapo* (Boiss.), *A*. *cilicica* ((Antoine & Kotschy) Carrière), *A. cephalonica* (Loud.), and *A*. *bormuelleriana* (Mattf.) as outgroup, obtained from the study of Awad et al. (2014) produced on the same sequencer and using common Western Alps samples as reference for correctly assigning allele length. Tree topology was tested by performing 10,000 bootstrap tests (Felsenstein, 1985). Linear regressions of pairwise population statistics (both *F*_ST_ and *R*_ST_, Slatkin (1995)) on geographical distances were performed to test for the presence of a phylogeographic signal, checking whether *R*_ST_ remained higher than *F*_ST_ after permuting alleles sizes over allelic states (Hardy et al., 2003) using SPAGeDi 1.4 (Hardy & Vekemans, 2002)⁠. Geographic distances between populations were computed using geographic Distance Matrix Generator version 1.2.3 (Ersts, American Museum of Natural History, Center for Biodiversity and Conservation, http://biodiversityinformatics.amnh.org/open_source/gdmg).

#### 2.3.3 Effective migration surface

Spatial population structure in the Pyrenees was explored by estimating effective migration surfaces (EEMS; Petkova et al., 2015). The EEMS method approximates a demographic model which evolves under equilibrium in time (isolation‐by‐distance) based on the stepping‐stone model (Kimura & Weiss, 1964). EEMS allows to test for deviations from isolation‐by‐distance and to test whether the relationship between genetic diversity and geography differs among habitats due to a combination of environmental and historical factors (gene flow barriers and/or admixture zones). We modeled the forty‐four Pyrenean populations on a dense regular grid, with migration between neighboring demes, and each population belonging to a different deme. Preliminary runs were made to obtain acceptance proportions of the MCMC between 20%–50%. Fine‐tuning was performed by modifying the proposed variances as follows: mSeedsProposalS2 = 0.05, qSeedsProposalS2 = 0.01, mEffctProposalS2 = 2.5, qEffctProposalS2 = 0.002, and mrateMuProposalS2 = 0.05. Since the choice of the grid could influence results, we followed the authors' instructions by averaging the estimates over two different grids (200 and 300 demes). For each, we performed three analyses, each with a different random seed. All EEMS analyses were run for 10 million iterations, sampling every 10,000 iterations with a burn‐in of 500,000. The six runs were combined and graphs were made using the accompanying R package rEEMSplots (http://www.github.com/dipetkov/eems).

#### 2.3.4 Blind approach to explore population genetic structure

We automatized runs of Structure 2.3.4 (Pritchard et al., 2000) using StrAuto (Chhatre & Emerson, 2017) to infer population structure and to assign individuals to populations. Due to possible gene flow between populations, we modeled (i) ancestry assuming that individuals have a mixed ancestry and (ii) allele frequencies assuming that frequencies in the different populations are likely to be correlated. Considering the number of genetic groups already observed in silver fir (Liepelt et al., 2009; Matías et al., 2016), the number of clusters expected in this study was three (Alps, Western Pyrenees, and Eastern Pyrenees); however, we tested the possibility of having an under‐structuring and therefore estimated the probability of observing from *K* = 1 to *K* = 8 clusters. For each value of K, we performed ten independent iterations with a burn‐in of 50,000 Markov chain Monte Carlo (MCMC) steps, followed by a run of 500,000 steps. We used Structure Harvester 0.6.94 (Earl & vonHoldt, 2012) and Clumpak (Kopelman et al., 2015) to compile and visualize results. To infer the most likely number of genetic clusters, we selected the value of K which gave the highest peak for Δ*K* (Evanno et al., 2005) and checked that the curve of the likelihood for this value of *K* reached a plateau as suggested by Pritchard et al. (2000).

#### 2.3.5 Demographic history

**FIGURE 1:**
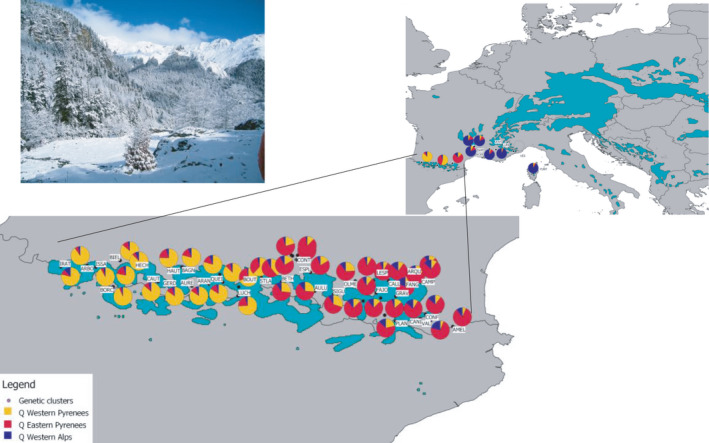
Location of the 52 silver fir populations sampled at the western edge of the geographic distribution of *Abies alba* (Mill.) in Europe and their membership to the three genetic clusters identified. The blue‐green area represents the geographic distribution of silver fir (see http://www.euforgen.org/species/abies‐alba/)

To infer the demographic history of silver fir, we compared scenarios using the coalescent/approximate Bayesian computation (ABC) framework implemented in DIYABC 2.1.0 (Cornuet et al., 2010) and both nuSSR and cpSSR data. To remove biases caused by gene flow between populations (which DIYABC cannot model), we only modeled the population having the highest ancestry coefficient in each cluster identified by the STRUCTURE analysis: Borc for the Western Pyrenees (*Q* = 0.91); Lesp for the Eastern Pyrenees (*Q* = 0.88); and Vent for the Southern Alps (*Q* = 0.93). An alternative sampling scheme would have been to pool a subset of individuals across populations belonging to the same genetic cluster, but this could have impacted the detection and the quantification of population size changes (Chikhi et al., 2010). In addition, we included a population (Stla, *Q* = 0.44) representing the contact zone between the genetic lineages in the Pyrenees. To confirm that the results were not biased by our choice of populations, we duplicated the analysis by choosing another set of 4 populations: Issa (*Q* = 0.92), Bele (*Q* = 0.87), Sigu (*Q* = 0.60), and Punt (*Q* = 0.86) for the Western Pyrenees, Eastern Pyrenees, admixed Pyrenees and Southern Alps, respectively (Figure 1).

**FIGURE 2:**
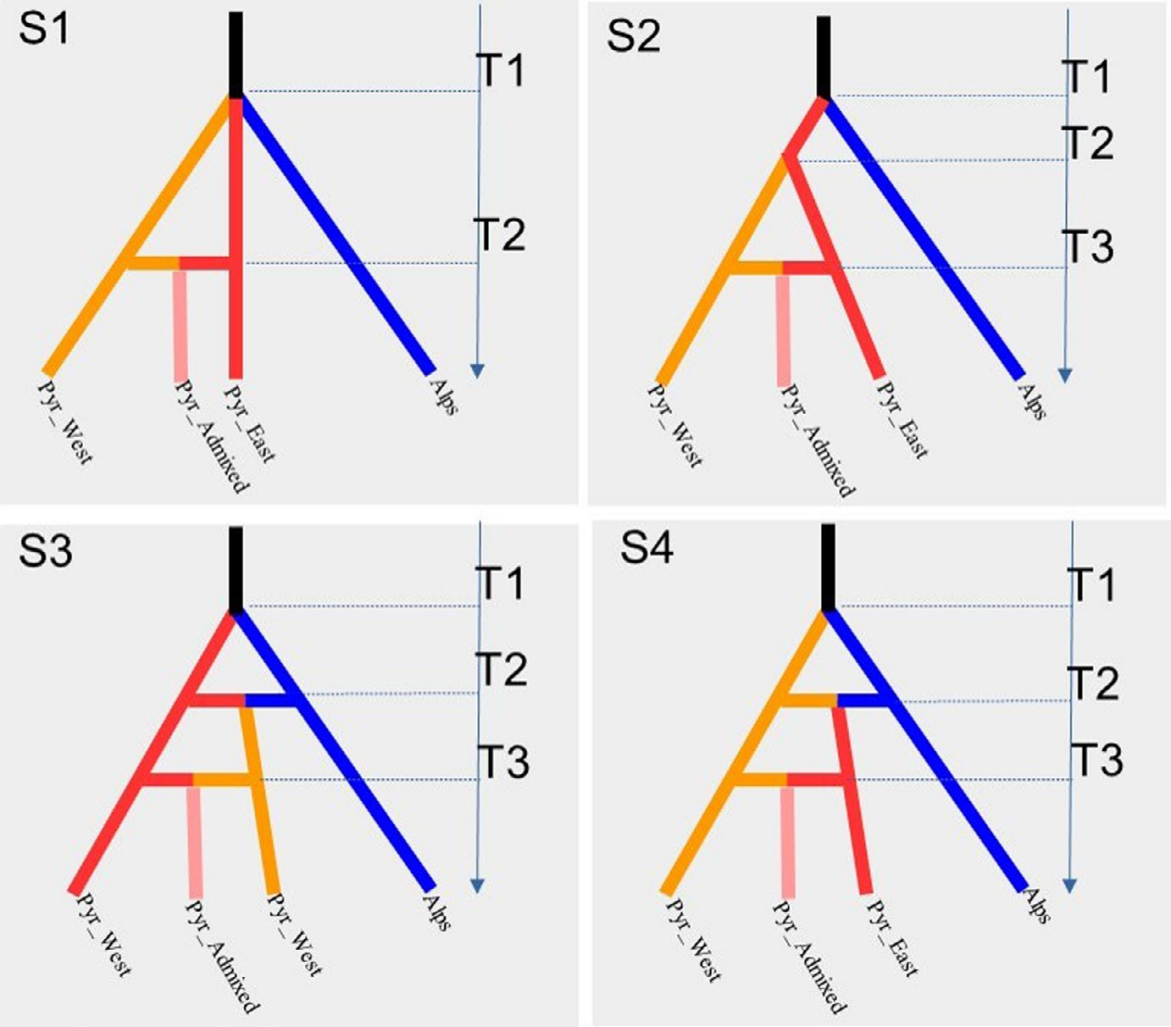
Demographic scenarios tested to infer past divergence events between four populations of silver fir. Four demographic scenarios are tested: Scenario S1 (Simple split), Scenario S2 (Hierarchical split), Scenario S3 (Alps‐East Pyrenees Split followed by two admixtures), and Scenario S4 (Alps‐West Pyrenees Split followed by two admixtures). In the four scenarios, fluctuation of the population sizes was allowed along final branches. Additional details are given in the main text

In scenario S1 (Simple split), a single event of divergence took place at time T_1_ from a common ancestor to give rise to the Alps, the Western Pyrenees, and the Eastern Pyrenees lineages, followed by a later secondary contact at time T_2_ between the two lineages from the Pyrenees to give rise to the admixed lineage (Figure 2). In scenario S2 (Hierarchical split), two events of divergence were considered. The Alps and Pyrenees lineages diverged first at T_1_ followed by a divergence at T_2_ between the Western and the Eastern Pyrenees and then at T_3_ by a secondary contact between lineages from Pyrenees. In scenario S3 (Alps–Eastern Pyrenees Split followed by two admixtures), at T_1_ a first event of divergence gave rise to both the Alps and the Eastern Pyrenees lineages, then at T_2_ the two lineages came into contact to give rise to the Western Pyrenees lineage. At T_3_, a secondary contact took place between the Pyrenean lineages to give rise to the admixed population of the Pyrenees. Finally, scenario S4 (Alps–West Pyrenees Split followed by two admixtures) is as in S3, but the Alps and Western Pyrenees lineages diverged first and came into contact later to give rise to the Eastern Pyrenees population. In the four scenarios, fluctuation of population sizes was allowed along final branches (at time T_a_, T_b_, T_c_, T_d_ for the Alps, Eastern Pyrenees, Western Pyrenees, and admixed Pyrenees populations, respectively).

We defined the prior distributions of the demographic parameters based on results obtained in the literature. All details of our approach are explained in Table S3.1. Both nuSSRs and cpSSRs were simulated using the generalized stepwise mutation model (Estoup et al., 2002; Zhivotovsky et al., 1997). The mean mutation rate of nuSSRs (*µNu_SSR_*) was drawn from a log‐uniform distribution constrained between 10^–4^ and 10^–3^, while for cpSSR, *µCp_SSR_* was sampled between 10^–8^ and 10^–3^. Priors for times of demographic events were drawn from a normal distribution of mean equal to 25,000 and standard deviation equal to 12,500. Priors for demographic sizes were drawn from a normal distribution of mean equal to 5,000 and standard deviation of 2,500. We applied the procedure “evaluate scenario‐prior combination” (Cornuet et al., 2010), which is based on both a principal component analysis and a test of rank, to choose a subset of the available summary statistics, that did not under‐ or overestimate genetic distance in simulated data sets, compared to the empirical one. Eight summary statistics were chosen (four single‐population summary statistics, and four two‐population summary statistics, Table S3.2 in Appendix S3).

For each scenario, one million simulations were performed. The most‐likely scenario was selected according to the results of a polytomous weighted logistic regression (Beaumont, 2010). Due to the high number of summary statistics, we replaced them with components of a linear discriminant analysis. The most‐likely scenarios were considered successfully recovered if the maximum posterior probability of the scenario was outside the 95% confidence intervals estimated for all other candidate scenarios (Cornuet et al., 2008). Confidence in scenario choice was evaluated by calculating type I error (classification error) and type II error from 1,000 pseudo‐observed data sets selected within the 1% simulations closest to observed statistics for each scenario (Cornuet et al., 2010). The posterior distribution of demographic parameters was finally estimated for the best scenario using a linear regression on the 1% closest simulations and applying a *logit* transformation to parameter values. Since the effective population size N_e_ depends on an unknown mutation rate value, we compared demographic fluctuations across time between genetic clusters by estimating the posterior distribution of the ratio between the current and past population diversity parameters using the ratio = θ_0_/θ_1_, with θ_0_ = N_0_μ0 (present) and θ_1_ = N_1_μ1 (past) (Barthe et al. (2017). Thus, by assuming that the mutation rate is constant through time (μ0 = μ1), the estimate of the demographic size ratio is no longer dependent on the (unknown) value of the mutation rate.

Bias and precision of parameter estimations were computed (mean relative bias (MRB), relative root mean square (RMSE), 50% and 95% coverage and Factor 2). The goodness‐of‐fit of the combination of the best model and parameter posterior distributions was appreciated using the model‐checking procedure in which the observed values of four additional summary statistics (Table S3.2 in Appendix S3) are compared to the values obtained from 1,000 replicated data generated under the scenario which gave the best probability. We did not use the same summary statistics to avoid risk of overestimating the quality of the fit.

#### 2.3.6 Correlating between genetic diversity and environmental factors

Our goal here was neither to look for signatures of selection and adaptation, nor to reveal causal relationships between neutral genetic diversity and environmental variables. Instead, it was to test whether environmental characteristics in the Northern Pyrenees (mainly past and present climatic variables) could be used as a proxy to model the organization of the genetic diversity in the Northern Pyrenees.

Environmental characteristics were obtained for each Pyrenean population. Slope, altitude, latitude, longitude, and nineteen bioclimatic variables from contemporary periods (1970–2000) and older periods (Mid‐Holocene approx. 6,000 years before present (BP), Last Glacial Maximum approx. 22,000 years BP, Last interglacial approx. between 120,000 and 140,000 years BP) were extracted from the WorldClim database (Hijmans et al., 2005). The descriptions of the environmental variables are reported in Appendix S4, Table S4.1 and the values for each population in Table S4.2.

For each genetic variable (Appendix S1, Table S1.2, Table S1.3), Pearson correlations with environmental variables were computed by applying a local false discovery rate (lfdr, Fdrtool R package, Strimmer, 2008) equal to 0.05. The presence of multicollinearity was tested and confirmed by calculating the variance inflator factor (VIF, car R Package, Fox & Weisberg, 2018) and using a threshold of VIF > 10 to consider multicollinearity. Since all tested variables caused multicollinearity, we then applied a forward stepwise regression which prevents multicollinearity problems in generalized linear models. The Bayesian information criterion (BIC) was used to find the most parsimonious regression models able to explain the genetic variables. For each genetic variable to explain, the best explicative environmental variables were finally included in a linear model to estimate the adjusted R‐square of the regression model and to estimate the regression coefficient and the *p* value of each retained environmental variables.

Environmental variables and data, genetic diversity parameters and many method descriptions and parameters are available in the Appendices (S1 to S5). All appendices are deposited open access at the following address: https://doi.org/10.15454/X6ZJU0.

## 3 RESULTS

### 3.1 Genetic diversity

**FIGURE 3:**
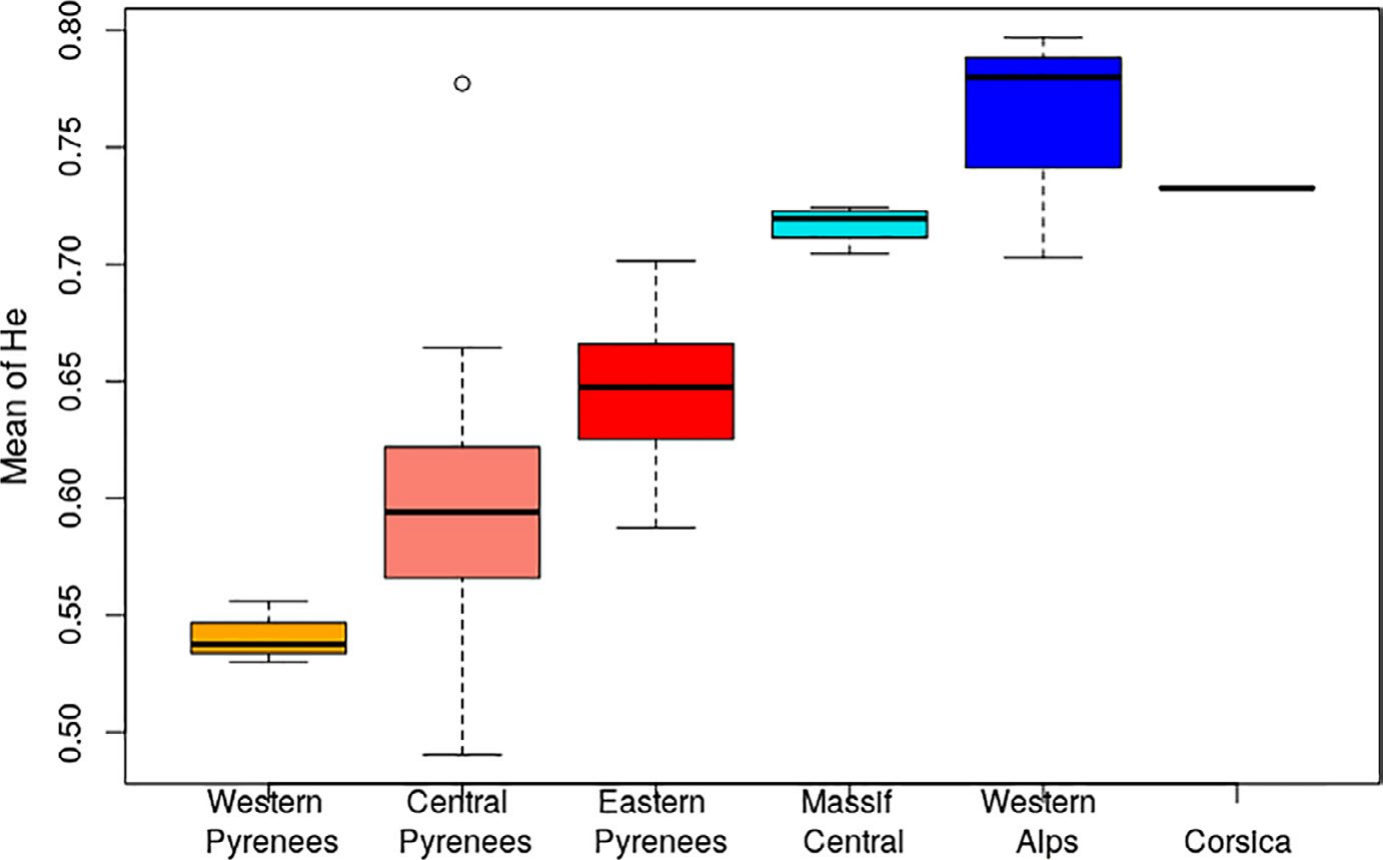
Distribution of mean heterozygosity (*H*_e_) at nuSSR in silver fir populations, by mountain range

The SF333 and SF50 nuSSR markers had a frequency of null alleles greater than 0.05 in more than half of the populations analyzed and were discarded. The combination of the three cpSSRs generated 72 haplotypes (eight variants for pt71936, 15 for pt30141, and three for ssr15169). The haplotype frequency distribution was L‐shaped, with a large number of low‐frequency haplotypes (60 with frequency lower than 0.01) and one common haplotype (frequency = 0.48). Population and mountain‐range genetic diversity levels are reported in Appendix S1, Table S1.3. Overall, genetic diversity was higher in Alpine populations (H_e_ at nuSSR between 0.72 and 0.74) than in the Pyrenees (0.55–0.65), while genetic diversity in the Pyrenees was highest in the eastern part of the range (Figure 3, Figure S1.1 in Appendix S1).

**FIGURE 4:**
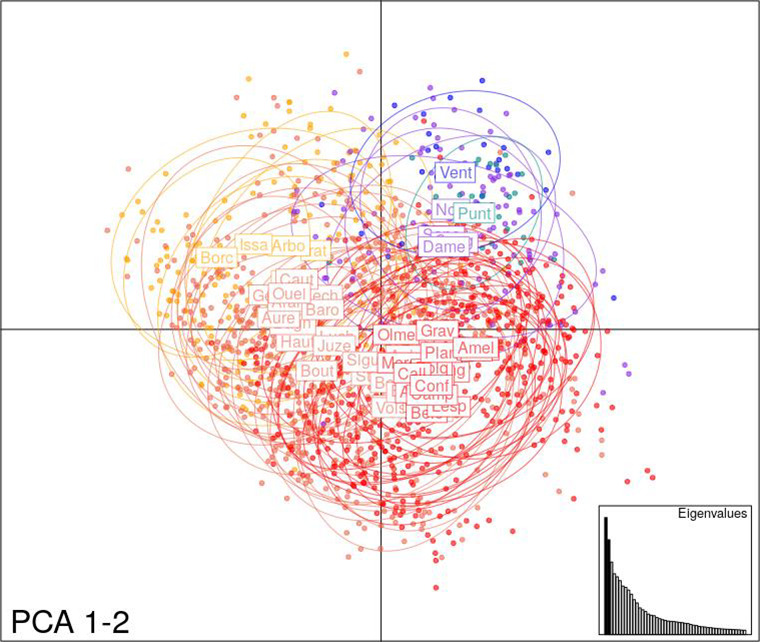
Principal component analysis (PCA) based on individual allelic frequencies at eight nuSSR and one cpSSR haplotype in silver fir populations sampled in six mountain ranges. Colors represent mountain ranges: Massif Central (purple), Western Alps (dark blue), Corsica (light blue), Western Pyrenees (orange), Central Pyrenees (peach), and Eastern Pyrenees (red). The code names of the populations are indicated in boxes at the center of gravity of their allelic distribution. The ellipses summarize the dispersion of the point cloud of each population with the center of the ellipse being the center of gravity of the population

Figure 4 displays the first two axes of an individual genotype‐based PCA. The first two axes of the PCA explain 16.4% of the total variance (9.1% and 7.3% for axes 1 and 2, respectively). The centers of gravity of the Western Alps, the Massif Central, and Corsica populations lie in the upper right quadrant of the PCA plot, while the centers of gravity of the Pyrenean populations are scattered throughout the remaining quadrants. Population and mountain‐range dot scatters overlap extensively, suggesting weak genetic differentiation.

Pairwise genetic differentiation (*F*_ST_, *R*_ST_) between mountain ranges was significant at both nuSSR and cpSSR and globally stronger between the Alps and the Pyrenees than among zones within the Pyrenees (Table S1.4 in Appendix S1). The *F*_ST_ matrix‐based phylogenetic tree had a deep split separating Alpine and Pyrenean populations (Figure S1.2 in Appendix S1). The Pyrenean branch had a ladder shape, suggesting isolation‐by‐distance from east to west in the Pyrenees, as confirmed by the significant positive slope of the linear regressions of pairwise statistics (both *F*_ST_ and *R*_ST_) on geographical distances. However, there was no significant difference between the slopes of the linear regressions of pairwise statistics based on allele identity (*F*_ST_) and allele size (*R*_ST_) meaning that populations could have been isolated for a relatively short period of time (in comparison to the mutation rate) or that gene flow between populations was strong. The EEMS confirmed the progressive colonization of the Pyrenees. However, in some parts, the presence of deviations from the step‐by‐step model was observed as either a stronger restriction of gene flow or an excess of gene flow (Appendix S1 ‐ Figure S1.3).

### 3.2 Genetic cluster inference

**FIGURE 5:**
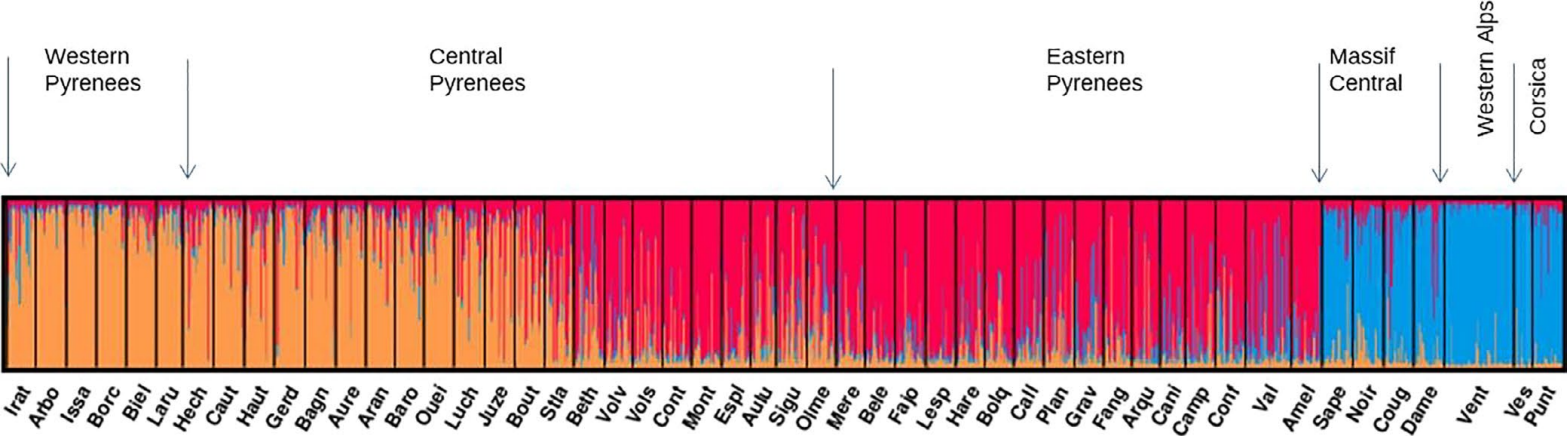
Structure analysis of 51 silver fir populations sampled in six mountain ranges in France. Each vertical bar represents a silver fir tree, organized by the longitude of the sampled populations in Western Pyrenees, Central Pyrenees, Eastern Pyrenees, Massif Central, Western Alps, and Corsica. Colors represent group membership. The proportion of each color in a bar represents the probability of assignment of the individual to each of the groups. Details of the populations are given in Appendix S1 Table S1.1

The most likely number of genetic clusters among all sampled populations was *K* = 3 (delta*K* = 247) (Figure S2.1a in Appendix S2). Two geographically distinct genetic clusters were detected in the Pyrenees (Western and Eastern Pyrenees clusters) while one cluster grouped the Alpine populations (Figure 5). Western Alps populations were more genetically homogeneous than populations from Massif Central or from Corsica, which had respectively 15% and 10% of individuals admixed with the Pyrenees lineage (vs. 0% in Western Alps). Western and Eastern Pyrenees samples belonged to two distinct ancestral parental populations, whereas Central Pyrenees populations (Juze, Bout, Stla, Beth) were split between the two clusters with a high degree of admixture. The two‐cluster genetic structure of the Pyrenees was confirmed when the Alpine populations were removed from the analysis (Figures S2.1b and S2.2 in Appendix S2).

### 3.3 Demographic history

Evaluations of the simulated data sets to test for the scenarios of divergence are provided in Appendix S3, Figure S3.1. The differences between the probabilities associated with each scenario were small, even for with scenario S2 the most probable (Figure S3.2, in Appendix S3), but only slightly more supported than the others. Redoing the analysis using a different set of four populations confirmed our first results (Figure S3.5, in Appendix S3). Further support to scenario S2 came from the phylogenetic tree obtained using *F*_ST_ values (Figure S1.2 in Appendix S1). However, the demographic inference lacked both accuracy and power, with a high posteriori error rate (*p* = .69) for all four scenarios. For scenario S2, type I error (rejection of S2 when true) and the type II error (acceptation S2 when not true) were respectively 0.56 and 0.65. Model adequacy of the best scenario S2 is nonetheless acceptable since among thirty tests, only four observed summary statistics (the mean genic diversity between pairs of Pyrenees clusters) deviated significantly from its simulated distribution (*p*‐value < .05; Table S3.7 in Appendix S3). Parameter estimates were also reliable with a relative median of the absolute errors ranging from 0.185 to 0.416 (Table S3.8 in Appendix S3).

Considering that the four scenarios gave similar estimates for temporal events (T1, T2, and T3), population size, admixture, and mutation rates (see Appendix S3, Tables S3.3 to S3.6), we conservatively decided to estimate model parameters as the mean of the medians of all scenarios. The 95% coverage of the posterior probability corresponds to the lowest and the highest limit obtained for the four scenarios. The Pyrenean and Alpine lineages diverged from an ancestral population 27,875 generations ago (95% confidence interval [CI] = 47,900–11,600). The Pyrenean lineage then separated 24,100 generations ago (95% CI = 39,300–12,900) into the Eastern and Western Pyrenean lineages. These came into contact 21,775 generations ago (95% CI = 37,900–11,400) to establish an admixture zone. Considering that silver fir is sexually mature at 30 to 40 years (Mauri et al., 2016), the mean of the medians for the divergence between the Alpine and the Pyrenean lineages was 836,250–1,115,000 years ago. The mean of the medians for the divergence between the two Pyrenean lineages was 723,000–964,000 years ago and the mean of the medians for their merging into a secondary contact zone located in the Central Pyrenees 653,250–871,000 years ago. For each of the four populations, we detected a synchronic population resizing 461,550–615,400 years ago. Thus, all major events of lineage divergence took place during the Mid‐Pleistocene Transition (0.7–1.25 million years ago), long before the Last Glacial Maximum (LGM) and the onset of the Holocene. All those estimates were confirmed by the second approximate Bayesian computing analysis using four different populations (Figure S3.5, Table S3.9 to S3.11).

**FIGURE 6:**
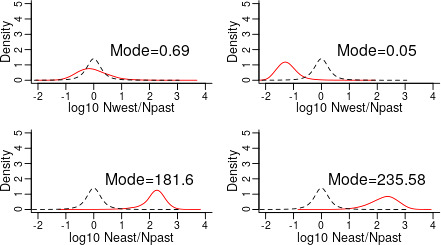
Comparison of the mode of the ratio between current and ancestral population sizes (*N*_current_/*N*_past_) in the western and eastern genetic clusters in French silver fir populations. Left columns: nuSSR; right column: cpSSR. The prior distribution is shown in gray dashed line, while the red dotted line is the posterior distribution. When the posterior distribution (in red) overlaps the prior distribution (mean of the prior = log_10 (Ncurrent/Npast)_ = 0, *that is*, the mode of the prior is 10^0^ = 1, that is, *N*_current_ = *N*_past_), we cannot exclude the hypothesis of a demographic equilibrium. This is the case in the Central Pyrenees (Pyr_C) at nuSSR and cpSSR. On the other hand, we can reject the equilibrium hypothesis when the mode of the posterior distribution is greater than 1 over 95% of its distribution. This is the case in the Eastern Pyrenees at both nuSSR and cpSSR where the ratio of current and ancestral population sizes is greater than zero, indicating a strong population expansion about 15,000 generations ago (see Table S3.4 for the timing of the event)

In the Eastern Pyrenees, the mode of posterior distribution of the ratio of current to past size (*N*_current_/*N*_past_) was greater than 1 over 95% of its distribution for both nuSSR and cpSSR (mode of *N*_current_/*N*_past_ = 182 and 235 for nuSSR and cpSSR, respectively) indicating a population expansion event (Figure 6). In contrast, in the Western Pyrenees, the *N*_current_/*N*_past_ ratio was smaller than 1 over 55% and 95% of its distribution for nuSSR and cpSSR respectively (modes of *N*_current_/*N*_past_ = 0.69 and 0.05 for nuSSR and cpSSR, respectively) indicating a population contraction event. In the Western Alps and the admixture zone of the Pyrenees (Figure S3.4 in Appendix S3), the results were less consistent between the two types of markers, and a demographic equilibrium (*N*_current_/*N*_past_ = 1) cannot be excluded from the 95% posterior distribution envelope.

### 3.4 Correlations between environmental variables and genetic parameters in the Pyrenees

The Pearson correlations gave 206 significant correlation tests with a local false discovery rate lower than 0.05 (Table S4.3 in Appendix S4). However, only 47 remained significant after the forward stepwise variable selection applied using a generalized linear model (Table S4.4 in Appendix S4). There were 12 significant correlations with precipitation seasonality (BIO15), nine with longitude, eight with mean temperature during the wettest quarter (BIO8), seven with mean temperature during the driest quarter (BIO9), four with temperature seasonality (BIO4), two with precipitations during the driest month (BIO14), and one significant correlation each with isothermality (BIO3), minimum temperature during the coldest month (BIO6), precipitations during the driest quarter (BIO17), precipitations during the warmest quarter (BIO18), and latitude. There was no significant difference (Pearson's chi‐squared test) between the number of significant bioclimatic explanatory variables related to temperature and those related to precipitation (21 and 16, respectively) and the absence of difference remained when nuSSR and cpSSR were considered separately. Considering the period, Pearson's chi‐squared tests indicated that cpSSR variables were more strongly associated with middle Holocene (6,000 years ago) bioclimatic variables, while the nuSSR variables were more strongly associated with last interglacial (LIG, 120,000 years ago) bioclimatic variables (Figure S4.4 in Appendix S4).

**FIGURE 7:**
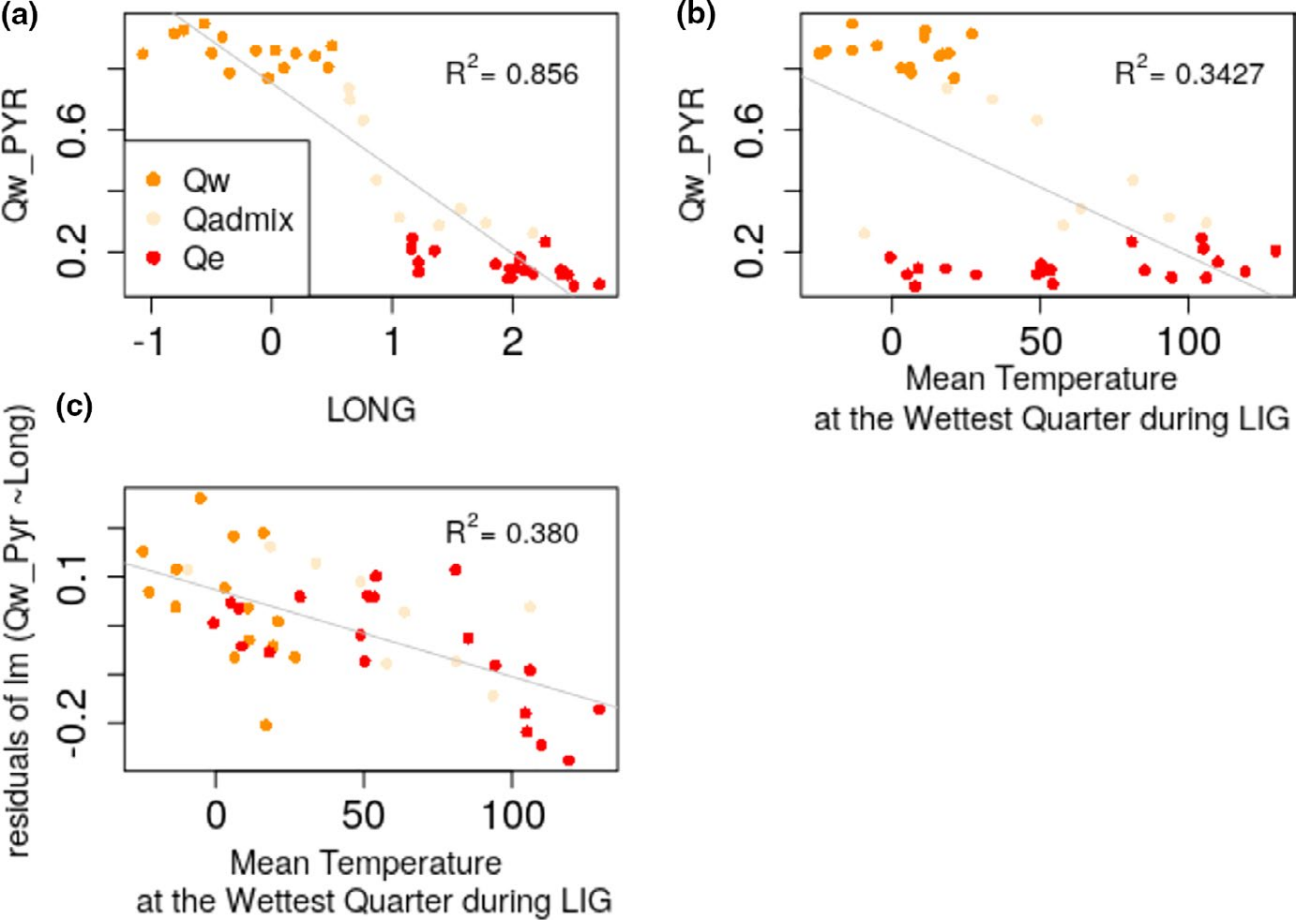
Plot of the regression of membership coefficient for the western genetic cluster *Q*_w_ in French silver fir populations according to the best explicative environmental variables. (a) 2D plot, *Q*_w_ according to the longitude (model 1). (b) 2D plot, *Q*_w_ according to the wettest quarter during LIG. (c) 2D plot, residual of the model 1 according to the mean temperature at the wettest quarter during LIG. Red dots are for values of *Q*_w_ > 0.75 and correspond to the western genetic cluster, dark orange dots for *Q*_w_ < 0.25 and correspond to the eastern genetic cluster, pale orange dots for 0.25 < *Q*_w_ < 0.75 and correspond to the admixed cluster

Among the significant regressions, high values of the membership coefficient for the western genetic cluster (*Q*_w_) were negatively associated with both longitude and mean temperatures during the LIG wettest quarter (Figure 7). Populations belonging to the western cluster (*Q*_w_ > 0.75) were associated with lower temperature (on average 3.94°C, standard deviation *SD* = 2.13°C), whereas the populations belonging to the eastern cluster (*Q*_w_ < 0.25) were associated with a larger range of temperatures (on average 6.3°C, *SD* = 4.02°C).

## 4 DISCUSSION

### 4.1 Past genetic divergence and demography of silver fir in the Pyrenees

The ABC‐based estimates of timing of divergence and effective population sizes should be considered as approximations since they are closely related to the mutation rate, unknown in silver fir. Here, we used published mutation rates from other plants as priors. Also, the main limitation of DIYABC is the assumed absence of migration among populations after they have diverged, which results in a probable under estimation of divergence time. In fact, our STRUCTURE analysis shows that admixture is still present in some easternmost and westernmost populations, outside of the admixture zone, indicating recent gene flow, either long distance or due to neighboring exotic plantation. Further, to convert generation time estimates into calendar time estimates, we assumed that sexual maturity is reached between 30 and 40 years in silver fir (Mauri et al., 2016), a reasonable approximation but subject to environmental variations. Finally, the use of only eight nuSSr and three cpSSR loci could explain in part why the very different scenarios of divergence we tested were not more strongly separated. While being aware of these limitations, the estimation of the demographic parameters remained consistent across the two repeated analyses and dated the origin of the two Pyrenean lineages to the middle Pleistocene, 0.7 M years ago, when Quaternary glacial cycle duration shifted from lasting an average of 41,000 years to an average of 100,000 years (Pisias & Moore, 1981).

Pyrenean lineages may have diverged from a single Alpine ancestor but also independently from two Alpine ancestral lines, as previously suggested for the evergreen shrub *Rhododendron ferrugineum* (Charrier et al., 2014). Silver fir was present between Eastern France (Jura) and the Pyrenees during the Pleistocene since pollen was detected in central France in the southern Loire river basin (Gauthier et al., 2005; Visset et al., 2003 and see Figure S5.1 in Appendix S5), but the lack of data from southwestern France makes it impossible to draw further conclusions.

Our results support the multiple refugia scenario, defined as “Refugia within Refugia” by Gómez and Lunt (2006) to explain biodiversity in the Iberian Peninsula. Even though we could not determine their exact location, the signal of a progressive colonization from east to west presupposes two glacial refugia: one for the eastern lineage, in the Eastern Pyrenees where the genetic diversity is highest, and a second more hypothetical refugium in the Central Pyrenees or in the pre‐Pyrenees chain as suggested for the perennial plant *Borderea pyrenaica* Miégev. (Segarra‐Moragues et al., 2007). The hypothesis of a possible refuge in the Western Pyrenees must be taken with caution, since it is not supported by fossil data. In addition, migrations during the Quaternary period led to secondary contact zones where the Eastern and Western Pyrenean lineages merged into admixed populations in the Central Pyrenees.

### 4.2 Distribution and environmental drivers of genetic diversity of silver fir in the Pyrenees

Our study shows that genetic diversity of silver fir decreases from east to west, Alpine populations being more diverse than populations in the Northern Pyrenees. This confirms the observations made on coarser sampling grids (Fady & Conord, 2010; Sancho‐Knapik et al., 2014; Vendramin et al., 1999). A decrease in genetic diversity between the Alps and the Pyrenees is also observed in several plants (Charrier et al., 2014; Despres et al., 2002) and animals (Caizergues et al., 2003). It is explained by harsher (colder and drier) climatic conditions on resident populations during the LGM in the west than in the east and/or long‐distance colonization during the Holocene or previous interglacials from favorable refugial habitats (Conord et al., 2012). This continental trend remains true at the Northern Pyrenees range scale for *Abies alba* and is probably explained by isolation‐by‐distance from east to west, as suggested by autocorrelation analyses, EEMS, and the historical demography signal (expansion in eastern and contraction in western populations). However, we also detected a contact zone allowing gene flow between genetic clusters in the Northern Central Pyrenees which could explain the absence of phylogeographic signal (absence of difference between *F*_ST_ and *R*_ST_) and the deviation from a stepping‐stone model in EEMS.

Silver fir displays a very clear genetic structure along the range of the Pyrenees as already observed in other plant and animal species (see references in the introduction), demonstrating the roles of east‐west land barriers in models of simple processes of differentiation, colonization, and mixing (Wallis et al., 2016). The geographical position of the Pyrenees between the Atlantic Ocean and the Mediterranean Sea, as well as the contrasted topography along the range (lower altitude in the west), resulted in the current contrasted regional climates but also in regional contrasts in glaciation patterns during the Quaternary periods (Calvet, 2004). This can explain why longitude is the environmental variable that explains most of the genetic diversity and structure in silver fir populations, dividing the Northern Pyrenees into two large blocks: the Western and the Eastern Pyrenees.

Additional factors can explain patterns of genetic diversity and in particular the distribution of ancestry coefficients. The relationships observed between the degree of membership to genetic clusters and the temperature during the wettest quarter at interglacial periods suggest a significant role of the past climate in the distribution of genetic diversity. Temperature during the wettest quarter is currently four degrees higher in the Eastern than in the Western Pyrenees (8°C vs. 4°C, Table S4.2), and it was five degrees higher during the last interglacial period, although mean temperature was much colder then, and probably more selective for trees (6.3°C vs. 1.2°C, Table S4.2). The strength of such environmental contrasts could have contributed to the maintenance of a strong differentiation between Pyrenean lineages. Whether long‐lasting differentiation in different environments has led to differential selection remains to be tested, but indications that regional environmental gradients lead to diversifying selection can be found in *Abies alba* studies from both the Spanish Pyrenees (Matías et al., 2016) and the Alps (Brousseau et al., 2016; Csilléry et al., 2020; Roschanski et al., 2016; Vitasse et al., 2009).

### 4.3 Conservation perspectives

The conservation of forests and forest habitats is part of the global strategy to conserve biodiversity, under the legal responsibility of each nation following the framework of the CBD (https://www.cbd.int/). Since 2016, recommendation WCC‐2016‐Rec‐104 of the International Union for Conservation of Nature (IUCN) “Integrating autochthonous forest genetic diversity into protected area conservation objectives” recommends that geographic entities that have *in situ* genetic diversity conservation as their main purpose should be recognized as protected areas (IUCN, 2016). Prioritization is essential to minimize biodiversity loss (Brooks et al., 2006), and in Europe, it is done under the guidance of EUFORGEN, the European Forest Genetic Resources Programme (http://www.euforgen.org/). Identifying which forests deserve the status of gene conservation unit (GCU) warrants the identification of well‐defined evolutionary lineages and the selection of forests where management is compatible with the occurrence of genetic processes governed by natural selection (Koskela et al., 2013; Lefèvre et al., 2020).

Major efforts are made in Europe for the conservation of the genetic resources of silver fir, with a total of 263 GCUs (http://www.euforgen.org/species/abies‐alba/), located in seven environmental zones (http://portal.eufgis.org), following the classification of Metzger et al. (2013) modified by de Vries et al. (2015). Seven GCUs currently represent the genetic diversity of the French Pyrenees, all analyzed in this study.

Four GCUs (the forests of Hares, Fanges, Arques, and Canigou) from the Eastern Pyrenees belong to the eastern lineage with a membership coefficient of 0.77 to 0.88. One GCU (the forest of Saint Lary) from the Central Pyrenees is admixed between the two lineages. The remaining two GCUs (the forests of Hautacam and Barousse), also from the Central Pyrenees, belong to the western lineage with a membership coefficient of 0.77 to 0.80. While the Eastern Pyrenees (in the geographical sense) and the eastern lineage are properly covered, with GCUs from forests at both low and high elevation, the Western Pyrenees (in the geographical sense) are not covered and the western lineage is poorly covered, with low elevation populations and the western margin of the distribution lacking. Geographical edges of distribution ranges demonstrate original local adaptations (Hampe & Petit, 2005; Parisod & Joost, 2010). Despite its low genetic diversity, the western margin of the geographical distribution of silver fir in the Pyrenees may harbor local adaptations that need further evaluation and protection.

Our study shows an absence of strict overlap between genetic clustering and geographic (topography and climate) structure in the Pyrenees and suggests that geography alone cannot be used as proxy for sampling for conservation. As genetic differentiation can occur over short distance along elevation gradients in silver fir (Csilléry et al., 2020; Roschanski et al., 2016), we advise that GCUs should be as large as possible (typically larger than 100 ha) to make gene flow possible across different elevations. In this perspective, including populations of admixed origin as GCUs (such as the Central Pyrenees Saint Lary forest) can significantly contribute to safeguarding evolutionary processes important for management under a changing climate, both for local resilience and as a resource for plantation forests (Hamilton & Miller, 2016; vonVoldt et al., 2017).

A remote and rather inaccessible area until recently, the Pyrenees represent one of the last refuges in Europe for some of its most spectacular and endangered mammals and birds, including the brown bear (*Ursus arctos arctos* L.), the Pyrenean ibex (*Capra pyrenaica pyrenaica* Schinz), and the bearded vulture (*Gypaetus barbatus* L.). Many strategies are developed to protect these species in the Pyrenees such as the strict protection of old‐growth silver fir, beech, and pine forests (Sabatini et al., 2018) under the Biodiversity Strategy of the European Union, the Natura 2000 network, national forest strategies, and regional initiatives. Creating and managing GCUs for silver fir and other forest tree species, maintaining their resilience, will further help conserve these and other mountain plant and animal species present in the Pyrenees.

## 5 CONCLUSION

Although much emphasis has been placed on the Holocene period, this study suggests that mid‐Pleistocene events have also durably shaped the current spatial structure of genetic diversity in natural forest tree populations. East‐west oriented mountain ranges, perpendicular to the main climate change directions of the Pleistocene, are suitable model systems to study the phylogeography and the demographic fate of mid‐ to high‐elevation mountain species, possibly emerging from different refugia. Geographical variables range‐wide remain insufficient and potentially misleading to capture patterns of genetic diversity for sampling with conservation purposes. Genetic studies are needed at the scale of a whole mountain range to allow the identification of all local particularities (e.g., in response to past environmental changes) for building and/or refining conservation strategies in mountain forest tree species.

## CONFLICT OF INTEREST

The authors have no competing interests to declare.

## AUTHOR CONTRIBUTION

**Caroline Scotti‐Saintagne:** Data curation (lead); Formal analysis (lead); Methodology (lead); Writing‐original draft (equal); Writing‐review & editing (equal). **Thomas Boivin:** Formal analysis (equal); Methodology (equal); Writing‐review & editing (equal). **Marie Suez:** Formal analysis (supporting); Methodology (supporting). **Brigitte Musch:** Conceptualization (equal); Funding acquisition (equal); Writing‐review & editing (equal). **Ivan Scotti:** Methodology (equal); Writing‐review & editing (equal). **Bruno Fady:** Conceptualization (lead); Funding acquisition (lead); Writing‐original draft (equal); Writing‐review & editing (equal).

## Supporting information

Appendix S1‐S5

## Data Availability

Genotypic data are archived and publicly accessible open access at: https://doi.org/10.15454/HVPAON.
