# Supplementary material for: Signature of mid‐Pleistocene lineages in the European silver fir (Abies alba Mill.) at its geographic distribution margin
Source: Ecol Evol. 2021 Jul 21;11(16):10984–99. doi: 10.1002/ece3.7886 (PMC8366861; doi:10.1002/ece3.7886)
Supplement: Supplementary file 1 — Appendix S1‐S5 [file ECE3-11-10984-s001.zip › Appendix_S1_Diversity_PhylogeoPyr_Aalba_final.docx]

***SUPPORTING INFORMATION***

**Signature of mid-Pleistocene lineages in the European silver fir (*Abies alba* Mill.) at its geographic distribution margin.**

***Appendix S1: Genetic diversity in silver fir***

**Figure S1.1:** Distribution of the mean of H_e_ according to the mountain range at three cpSSR

**Figure S1.2:** Unrooted Neighbor joining tree from the F_ST_ distance matrix between silver fir populations

**Figure S1.3:** Estimated Effective Migration Surfaces (EEMS) in the Pyrenees

**Table S1.1:** Location details of the sampled populations of silver fir.

**Table S1.2:** List and definition of the genetic parameters.

**Table S1.3:** Genetic diversity indices at nuSSR. cpSSR loci and cpSRR haplotypes in the sampled populations of silver fir (**see separate file: Appendix_S1_Table.xls**)

**Table S1.4:** Pairwise genetic differentiation between mountain ranges

**Appendix 1 - Figure S1.1**: Distribution of the mean of H_e_ in silver fir according to the mountain range at three cpSSR


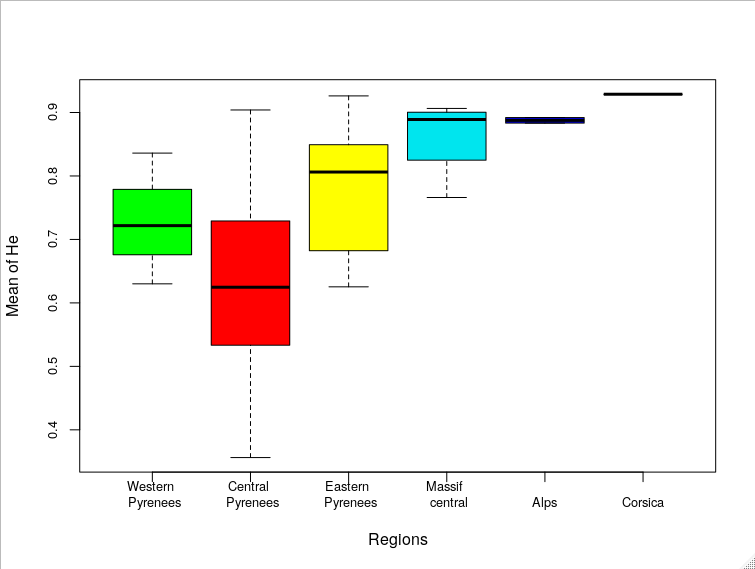


**Appendix S1 - Figure S1.2**: Unrooted Neighbor joining tree from the F_ST_ distance matrix between silver fir populations.


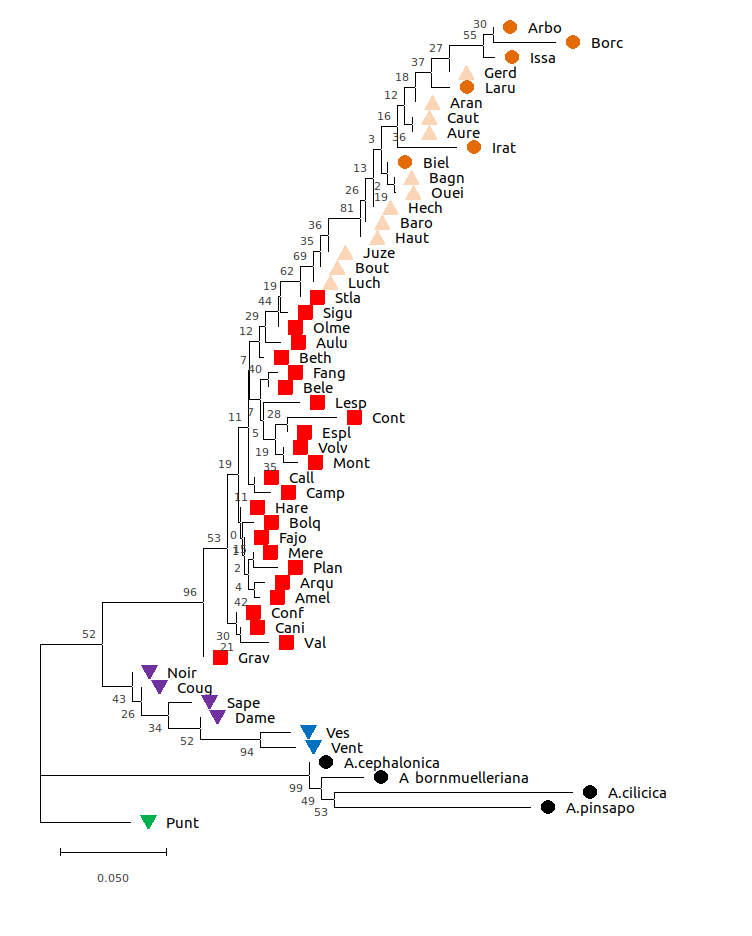


Outgroup

Corsica

Western Alps

Massif Central

Eastern Pyrenees

Central Pyrenees

Western Pyrenees

Node labels indicate the confidence of the branches after 10000 bootstrap re-samplings. The mountain ranges to which each population belongs is indicated by the tip marker (see legend). The full name of the populations is given in Appendix S1 - Table S1.1. Outgroups are genotypes of Mediterranean firs (Awad et al.. 2014).

The phylogenetic tree is composed of two main branches that separate the Alpine populations from the Pyrenean populations. The Pyrenean branch has a ladder shape suggesting isolation by distance from east to west.

Awad L. Fady B. Khater C. Roig A. Cheddadi R (2014). Genetic Structure and Diversity of the Endangered Fir Tree of Lebanon (Abies cilicica Carr.): Implications for Conservation. Plos One 9(2)

**Appendix S1 - Figure S1.3:** Estimated Effective Migration Surfaces (EEMS) in the Pyrenees


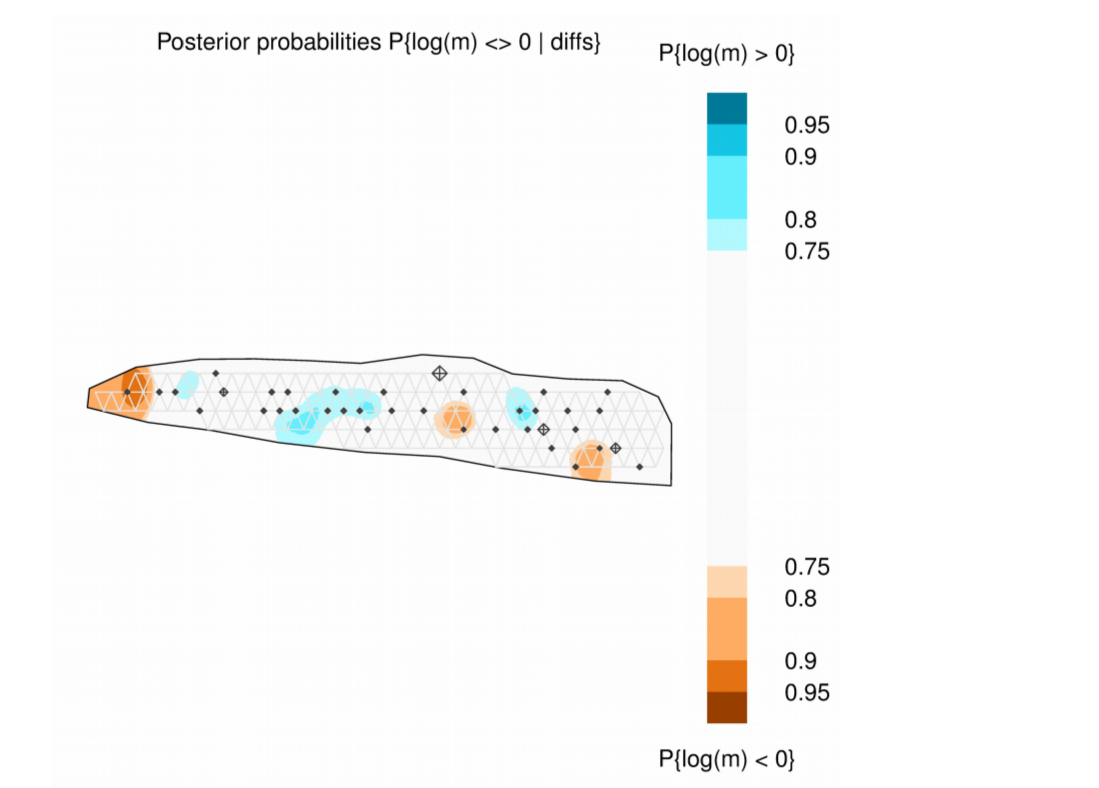


The colour contour plot was produced by averaging draws from the posterior distribution of the migration rates. interpolating between grid points. The parameter log(m) denotes the effective migration rate on a log10 scale. relative to the overall migration rate across the habitat. When P{log(m)<0) = 0. the effective migration rate is equal to the overall mean migration (stepping stone model). When P{log(m)<0) = 1. the effective migration is tenfold faster than the average (deviation to the SSM model likely due to gene flow). When P{log(m)<0) = -1. the effective migration is tenfold lower than the average (deviation to the SSM likely due to population isolation).

**Appendix S1 - Table S1.1:** Location details of the sampled populations of silver fir.

| **Mountain range** | **Pop label** | **Slope in %** | **Latitude** | **Longitude** | **Altitude** | **Name of community** | **Name of forest** | **Additional information. monitoring networks** |
| --- | --- | --- | --- | --- | --- | --- | --- | --- |
| PYR_W | Irat | 40 | 43.000317 | -1.0677833 | 1072 | Larrau | FC d’Iraty |  |
| PYR_W | Arbo | 35 | 43.013772 | -0.8140431 | 1178 | Sainte Engrâce | FC de Sainte Engrâce (bois d’Arbouty) |  |
| PYR_W | Issa | 35 | 42.997517 | -0.73105 | 1394 | Osse en Aspe | FS d’Issaux | Seed stand AAL601-015 |
| PYR_W | Borc | 50 | 42.80815 | -0.5623167 | 1362 | Borce | FC de Borce (lac d’Anglus) | Central zone of Pyrenees National Park |
| PYR_W | Biel | 25 | 43.061688 | -0.4957043 | 980 | Bielle | FC de Bielle et Bilhères |  |
| PYR_W | Laru | 50 | 42.96355 | -0.4069 | 1341 | Eaux-Bonnes | FS d’Assouste |  |
| PYR_C | Hech | 45 | 42.98587 | -0.3457215 | 1116 | Hèches | GF d’Hèches (Baronnies) |  |
| PYR_C | Caut | 60 | 42.85525 | -0.1276833 | 1456 | Cauterets | FS de la vallée de Saint-Savin | Central zone of Pyrenees National Park |
| PYR_C | Haut | 45 | 42.991726 | -0.0252794 | 1416 | Vic de Préchac | FS Hautacam | Gene conservation unit AA25 |
| PYR_C | Gerd | 70 | 42.811267 | 0.0318167 | 1521 | Gèdre | FC de Gèdre (sapinière de Barrada) | Pyrenees old growth network |
| PYR_C | Bagn | 25 | 42.997518 | 0.1005469 | 1369 | Bagnères de Bigorre | FC de Bagnères de Bigorre | Seed stand AAL601-014 |
| PYR_C | Aure | 45 | 42.8151 | 0.1985333 | 1578 | Aragnouet | FC d’Aragnouet (sapinière de Couplan) |  |
| PYR_C | Aran | 60 | 42.834467 | 0.3628833 | 1333 | Camparan | FC de Camparan | Seed stand AAL601-008 |
| PYR_C | Baro | 40 | 42.92875 | 0.4683539 | 1283 | Ferrère | FD de Barousse | Seed stand AAL601-007 and Gene conservation unit AA24 |
| PYR_C | Ouei | 65 | 42.8526 | 0.4981 | 1649 | Bourg d'Oueil | FD de Bourd d’Oueil |  |
| PYR_C | Luch | 70 | 42.718972 | 0.6445389 | 1459 | Bagnères de Luchon | FC de Bagnières de Luchon |  |
| PYR_C | Juze | 60 | 42.806583 | 0.65145 | 1385 | Juzet de Luchon | FD de la Cigalère | Seed stand AAL601-002 |
| PYR_C | Bout | 15 | 42.908234 | 0.7604193 | 1449 | Boutx | FC de Boutx | Silvapyr plot 31C1N |
| PYR_C | Stla | 55 | 42.895033 | 0.8712833 | 1417 | St Lary | FD de St Lary | Gene conservation unit AA12 |
| PYR_C | Beth | 55 | 42.856633 | 1.0649333 | 1621 | Bethmale | FD de Bethmale |  |
| PYR_C | Volv | 30 | 43.112846 | 1.1619892 | 348 | Ste Croix Volvestre | FD Ste Croix Volvestre | Recent forest |
| PYR_C | Vols | 30 | 43.106481 | 1.1722921 | 398 | Ste Croix Volvestre | FD Ste Croix Volvestre | Old growth forest |
| PYR_C | Cont | 40 | 43.066762 | 1.2182454 | 462 | Contrazy | Forêt de M. Naudin |  |
| PYR_C | Mont | 35 | 43.111882 | 1.2208485 | 456 | Montbrun Bocage | Forêt de M. Lannes |  |
| PYR_C | Espl | 70 | 42.946358 | 1.3460861 | 953 | Esplas de Sérou | FD de Belissens | Silvapyr plot 09C1R |
| PYR_C | Aulu | 65 | 42.76699 | 1.3868552 | 1358 | Aulus les Bains | FC d’Aulu les Bains |  |
| PYR_C | Sigu | 60 | 42.733467 | 1.5667 | 1361 | Siguer | FD du Val de Siguer |  |
| PYR_C | Olme | 47 | 42.864393 | 1.7790294 | 1457 | Montferrier | FC de Montferrier |  |
| PYR_E | Mere | 30 | 42.669867 | 1.8550821 | 1679 | Mérens | FD de la Haute-Ariège |  |
| PYR_E | Bele | 30 | 42.866567 | 1.9563531 | 915 | Bélesta | GF de Cabrefol | Seed stand AAL361-004 |
| PYR_E | Fajo | 60 | 42.748364 | 1.9811668 | 1443 | La Fajolle | FD de la Fajolle | Seed stand AAL361-006 |
| PYR_E | Lesp | 20 | 42.913217 | 1.98515 | 547 | Lesparrou | GF de Vilhac |  |
| PYR_E | Hare | 50 | 42.699017 | 2.0243333 | 1587 | Mijanès | FD des Hares | Gene conservation unit AA11 |
| PYR_E | Bolq | 50 | 42.53335 | 2.0635833 | 1715 | Bolquère | FC de Bolquère | Seed stand AAL361-007 |
| PYR_E | Call | 20 | 42.866052 | 2.1007247 | 972 | Coudons | FD Callong-Mirailles | RENECOFOR plot SP 11/95 and seed stand AAL361-001 |
| PYR_E | Plan | 60 | 42.476117 | 2.1688167 | 1774 | Planès | FC de Planès. |  |
| PYR_E | Grav | 5 | 42.71505 | 2.1706 | 1592 | Le Bousquet | FD de Montnaie-Gravas |  |
| PYR_E | Fang | 10 | 42.832992 | 2.2749935 | 896 | Lapradelle | FD des Fanges | Gene conservation unit AA07 |
| PYR_E | Arqu | 55 | 42.925936 | 2.398654 | 724 | Arques | FD du Rialsesse et FC d’Arques | Gene conservation unit AA06 |
| PYR_E | Cani | 47 | 42.500995 | 2.405717 | 1659 | Casteil | FD du Canigou | Gene conservation unit AA08 |
| PYR_E | Camp | 47 | 42.885261 | 2.4206547 | 791 | Camps sur l'Agly | FD de l’Eau salée |  |
| PYR_E | Conf | 55 | 42.543833 | 2.45905 | 1800 | Taurinya | FD du Canigou |  |
| PYR_E | Val | 60 | 42.482423 | 2.508065 | 1475 | Corsavy | FD du Haut Vallespir (sapinière du Riuferrer) |  |
| PYR_E | Amel | 55 | 42.423733 | 2.7215667 | 1308 | Amélie les Bains | FP de la Chaîne Thermale du soleil | Central zone of Cévennes National Park |
| MC | Sape | 62 | 44.470261 | 3.6010845 | 1152 | Lanuéjols | FD du Sapet | Central zone of Cévennes National Park |
| MC | Noir | 57 | 44.440673 | 3.8414454 | 1202 | Altier | FD du Bois Noir | Central zone of Cévennes National Park |
| MC | Coug | 28 | 44.425841 | 3.8699799 | 1367 | Altier | FD du Mont Lozère | Central zone of Cévennes National Park |
| MC | Dame | 50 | 44.394581 | 3.8989852 | 1344 | Pourcharesses | Forêt du pré de la Dame | Central zone of Cévennes National Park |
| WS | Vent | - | 44.180004 | 5.246604 | 1374 | Beaumont du Ventoux | FC de Beaumont du Ventoux | Gene conservation unit AA22 |
| WS | Ves | - | 43.97074 | 7.36577 | 1600 | Saint Martin Vésubie | FC de Saint Martin Vésubie |  |
| C | Punt | 40 | 41.98568 | 9.11203 | 1520 | Ciamannacce | FT Punteniellu | Gene conservation unit AA04 |
| WS | Lure | - | 44.1212 | 5.8241 | 1475 | Valbelle | FD de Lure |  |

Mountain range: PYR_E for Eastern Pyrenees. PYR_W for Western Pyrenees. Pyr_C for Central Pyrenees. MC for Massif Central. WA for Western Alps. C for Corsica.

Name of forest: FC for communal forest. FD for state forest. FS for multi-community forest. FP for private forest. GS for multi-owner forest

**Table S1.2:** List and definition of the genetic parameters used in the analyses

| **Genetic variables** | **Genetic markers** | |
| --- | --- | --- |
| Number of alleles (N_A_) | Nussr | CpSSR. cpHaplotype |
| Number of effective alleles (N_AE_) | Nussr | CpSSR. cpHaplotype |
| Expected heterozygotie (H_E_) | Nussr | CpSSR. cpHaplotype |
| Number of rarefied alleles (N_A50_.N_A22_ ) | Nussr | CpSSR. cpHaplotype |
| Proportion of unique allele (A_u_) | Nussr | CpSSR. cpHaplotype |
| Presence/Absence of private Allele (P) | Nussr | CpSSR. cpHaplotype |
| Number of rarefied private alleles (P_50_. P_22_ ) | Nussr | CpSSR. cpHaplotype |
| Inbreeding coefficient (F_I_) | Nussr |  |
| The averaged membership coefficient Q | Nussr |  |

**Appendix S1 - Table S1.4:** Pairwise genetic differentiation between mountain ranges.

F_ST_ below the diagonal and R_ST_ above the diagonal (blue font). Genotypic differentiation pairs were tested using an exact G test (Fisher's method) across all loci. P-value was < 0.001 for all pairs.

A. nuSSR

|  | Western Pyr | Central  Pyr | Eastern  Pyr | Massif  Central | Western  Alps | Corsica |
| --- | --- | --- | --- | --- | --- | --- |
| Western Pyr |  | 0.03 | 0.10 | 0.16 | 0.30 | 0.17 |
| Central Pyr | 0.01 |  | 0.05 | 0.13 | 0.30 | 0.15 |
| Eastern Pyr | 0.10 | 0.05 |  | 0.07 | 0.23 | 0.12 |
| Massif Central | 0.13 | 0.12 | 0.09 |  | 0.05 | 0.03 |
| Western Asps | 0.19 | 0.19 | 0.17 | 0.04 |  | 0.04 |
| Corsica | 0.19 | 0.18 | 0.15 | 0.08 | 0.01 |  |

B. cpSSR

|  | Western Pyr | Central  Pyr | Eastern  Pyr | Massif  Central | Western  Alps | Corsica |
| --- | --- | --- | --- | --- | --- | --- |
| Western Pyr |  | 0.02 | 0.00 | 0.10 | 0.33 | 0.09 |
| Central Pyr | 0.01 |  | 0.04 | 0.21 | 0.47 | 0.22 |
| Eastern Pyr | 0.02 | 0.03 |  | 0.08 | 0.29 | 0.06 |
| Massif Central | 0.07 | 0.13 | 0.04 |  | 0.07 | 0.01 |
| Western Alps | 0.17 | 0.25 | 0.12 | 0.02 |  | 0.11 |
| Corsica | 0.09 | 0.14 | 0.06 | 0.05 | 0.10 |  |
