# Supplementary material for: Signature of mid‐Pleistocene lineages in the European silver fir (Abies alba Mill.) at its geographic distribution margin
Source: Ecol Evol. 2021 Jul 21;11(16):10984–99. doi: 10.1002/ece3.7886 (PMC8366861; doi:10.1002/ece3.7886)

***SUPPORTING INFORMATION***

**Signature of mid-Pleistocene lineages in the European silver fir (*Abies alba* Mill.) at its geographic distribution margin.**

**Appendix S2: Genetic cluster analysis in silver fir**

**Figure S2.1:** Results of the blind approach using STRUCTURE. Likelihood of K and Delta K plots. **a.)** at the global scale (from Alps to Pyrenees). **b.)** at the scale of the Pyrenees.

**Figure S2.2**: Barplot of ancestry proportions for genetic clusters averaged over five runs for the best likely number of clusters (K).

**Appendix S2, Figure S2.1 a:** Results of the unbiased genetic diversity structuring approach using STRUCTURE. Likelihood of K and Delta K plots at the global scale (from Alps to Pyrenees).

Likelihood of K and Delta K were calculated as in Evanno et al. (2005) and implemented in Structure harvester by Earl & vonHoldt (2012). See Material and Methods.


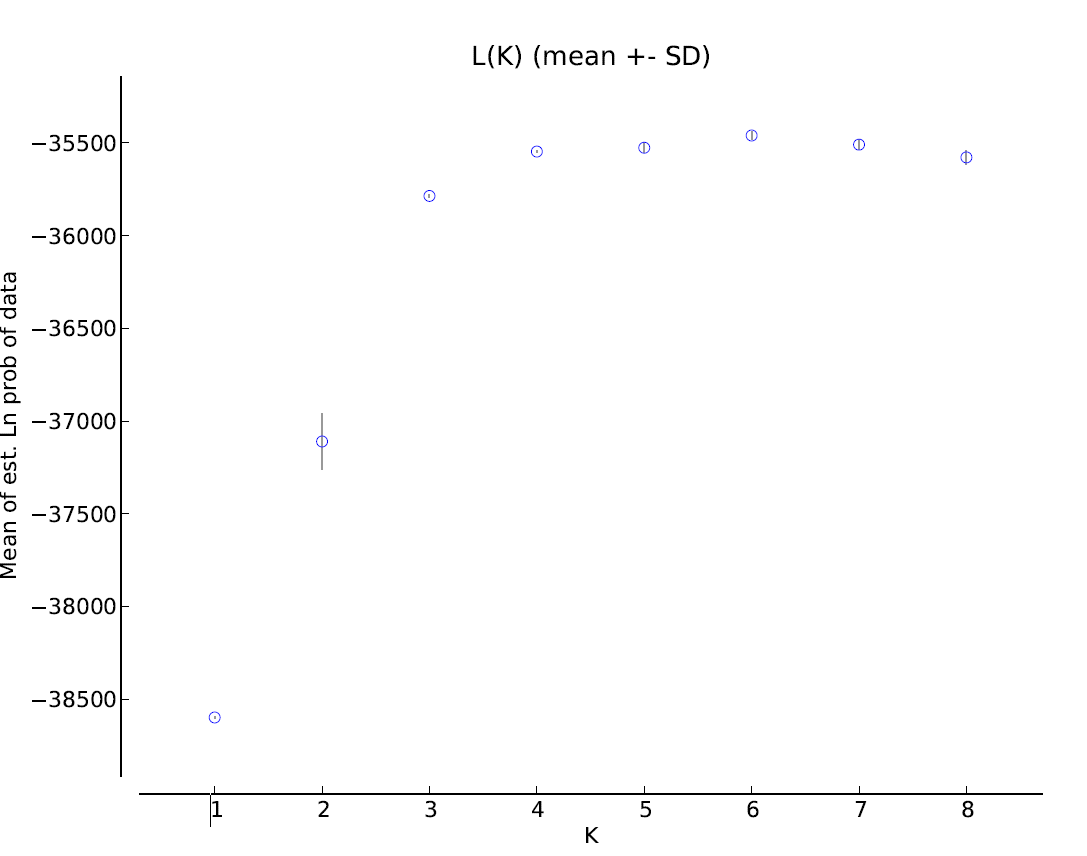


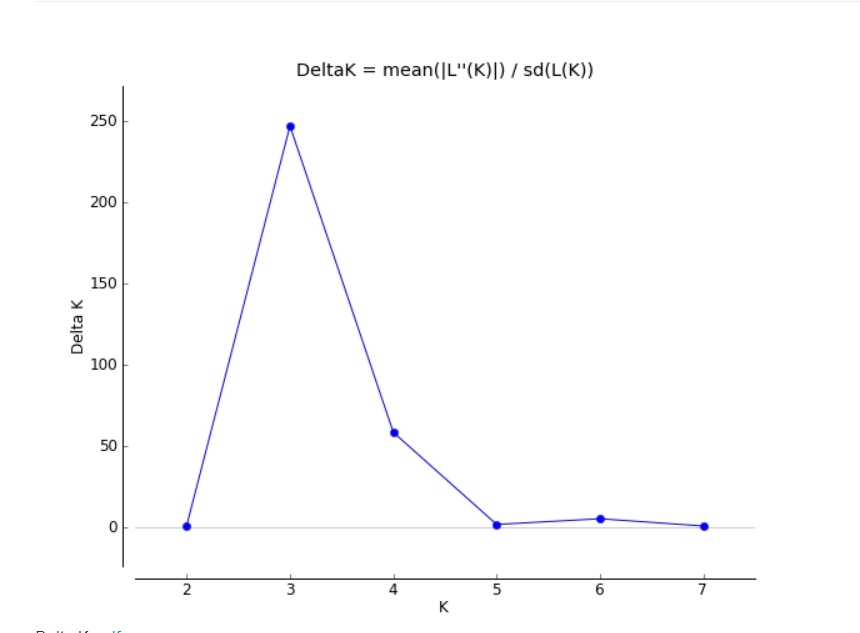


**Appendix S2, Figure S2.1 b:** Results of the unbiased genetic diversity structuring approach using STRUCTURE. Likelihood of K and Delta K plots at the Pyrenees scale.

Likelihood of K and Delta K were calculated as in Evanno et al. (2005) and implemented in Structure harvester by Earl & vonHoldt (2012). See Material and Methods.


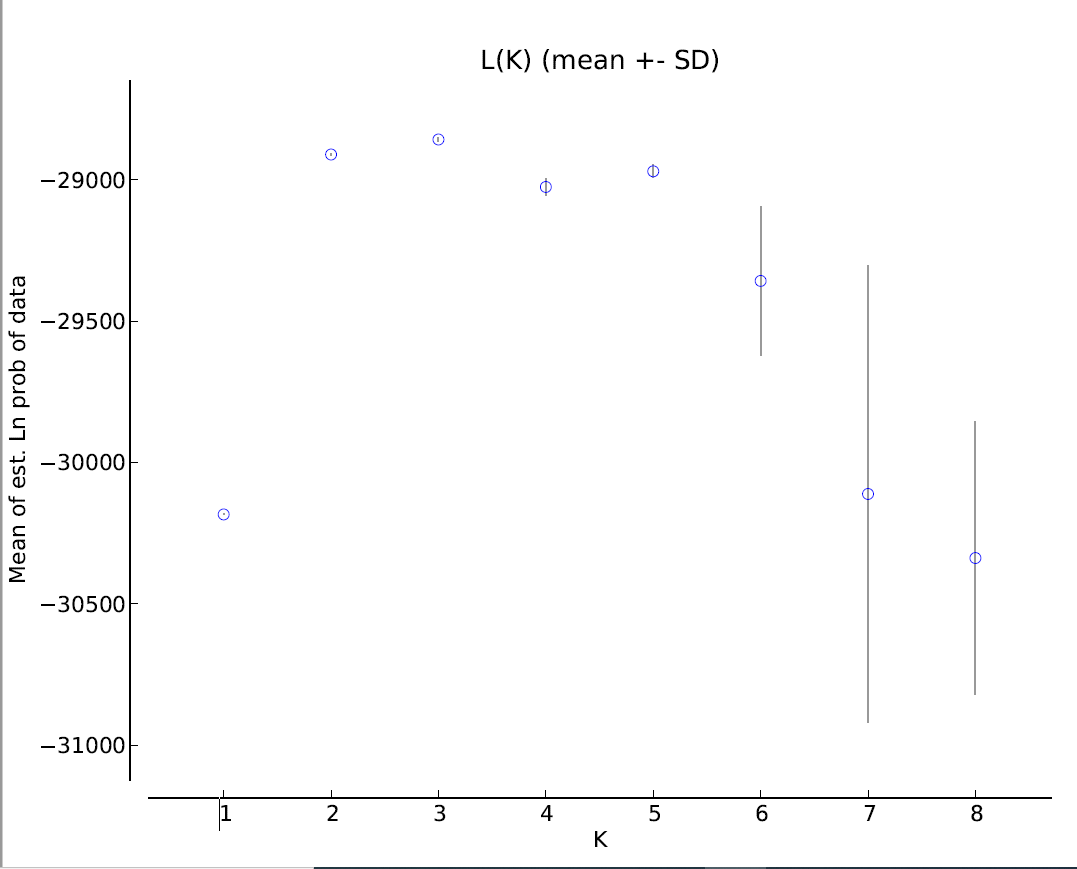


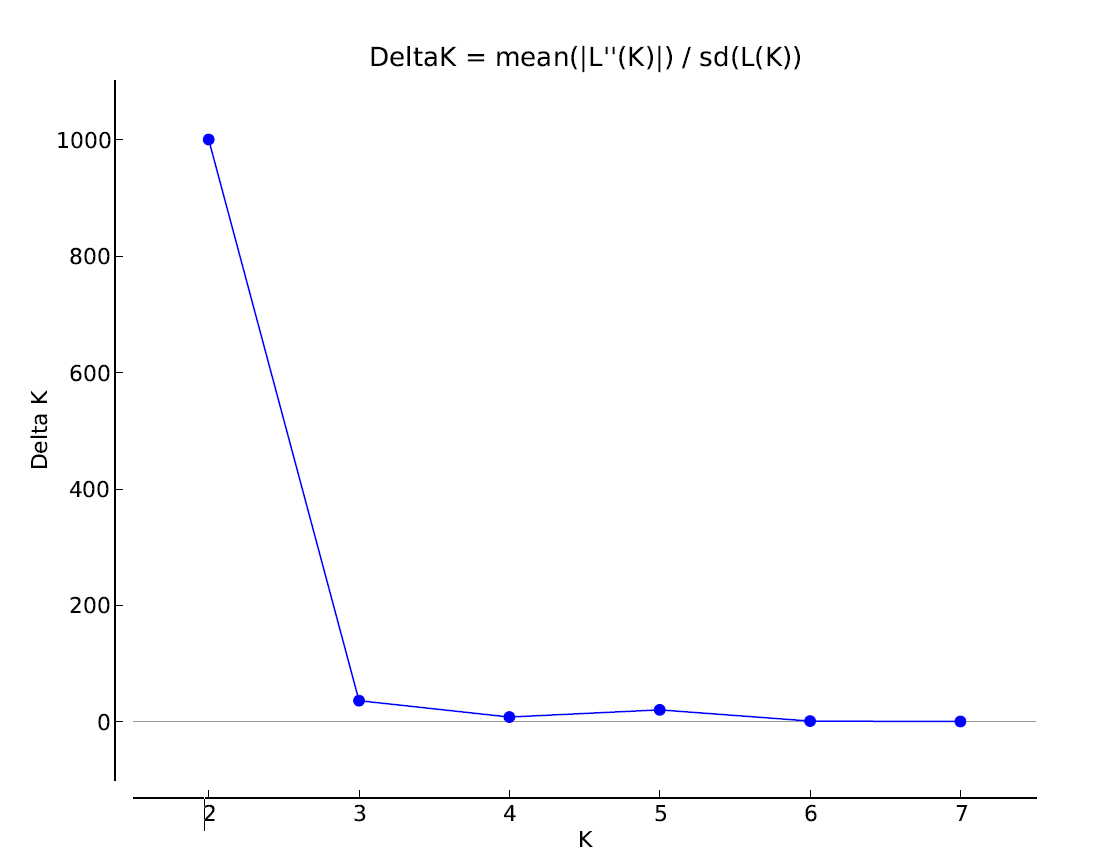

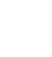

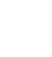


**Appendix S2, Figure S.2:** Barplot of ancestry proportions for genetic clusters averaged over five runs for the best likely number of clusters (K). At the scale of the Pyrenees, K = 2 is the most likely number of genetic clusters (see Figure S2.1 b). Each individual is represented by a vertical bar divided into two color segments representing the two gene pools identified by the STRUCTURE analysis. Population labels are as in Table S1.1


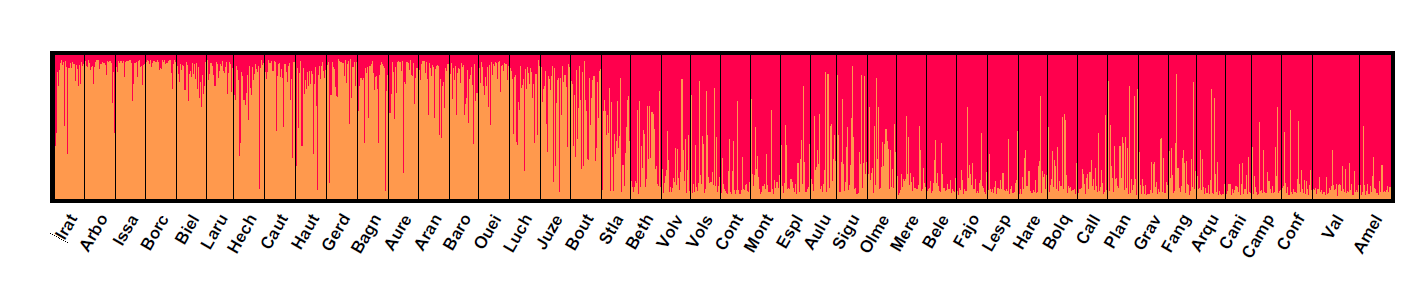

Supplement: Supplementary file 1 — Appendix S1‐S5 [file ECE3-11-10984-s001.zip › Appendix_S2_Clustering_analysis_PhylogeoPyr_Aalba_final.docx]
