# Supplementary material for: Signature of mid‐Pleistocene lineages in the European silver fir (Abies alba Mill.) at its geographic distribution margin
Source: Ecol Evol. 2021 Jul 21;11(16):10984–99. doi: 10.1002/ece3.7886 (PMC8366861; doi:10.1002/ece3.7886)
Supplement: Supplementary file 1 — Appendix S1‐S5 [file ECE3-11-10984-s001.zip › Appendix_S5_Fossil_PhylogeoPyr_Aalba_final.docx]

***SUPPORTING INFORMATION***

**Signature of mid-Pleistocene lineages in the European silver fir (*Abies alba* Mill.) at its geographic distribution margin.**

***Appendix S5: fossil pollen in silver fir***

Figure S1: location of French sites where fossil pollen of A*bies alba* was observed.

We extracted the data available for *Abies alba* in France from the European pollen data base (<http://www.europeanpollendatabase.net/fpd-epd/>). Both calibrated and non-calibrated data were extracted.

The map was drawn using the interface website (<https://umap.openstreetmap.fr/fr/map/fossil-pollen-of-abies-alba_454786#6/43.644/3.208>). Note that site location information is degraded.

Blue squares denote the presence of *A. alba* before 8,000 YBP (Years Before Present, i.e. the number of years before 1950); red pins: presence of *A. alba* after 5,0000 YBP (calibrated data); orange pins: presence of *A. alba* after 10,000 YBP (calibrated data); yellow pins: presence of *A. alba* after 10,000 YBP (non-calibrated data).


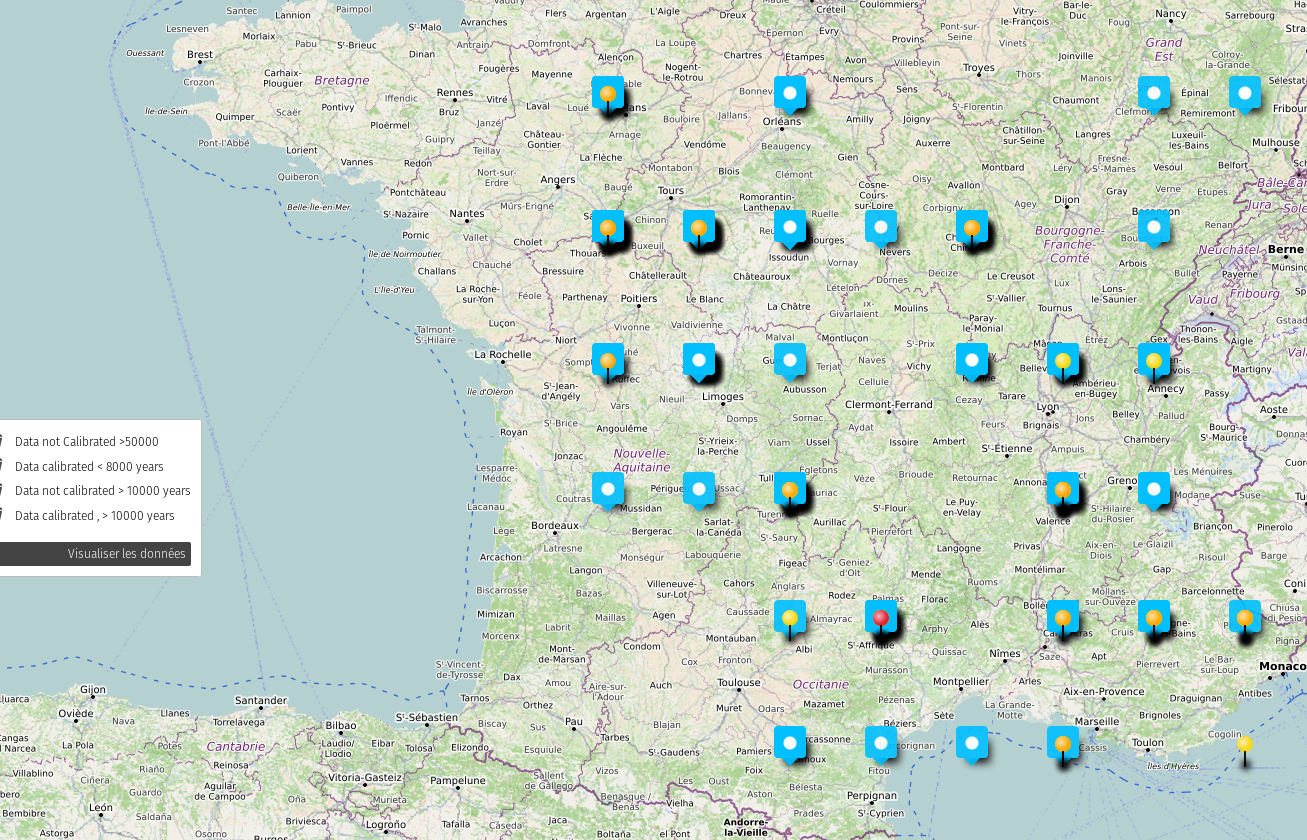


Data are lacking in the Pyrenees. However, *A. alba* was present during the Holocene in the Eastern Pyrenees, and during both the Holocene and the Pleistocene in the Massif Central as early as 120,000 YBP in the lac du Bouchet (J.L. de Beaulieu. 1988). *A. alba* was also present during the Pleistocene in the south of the Loire basin (Visset et al., 2003; Gauthier et al., 2005).

Visset, L., Cyprien, A.L., Carcaud, N., & Ouguerram, A. (2003). De la fin du Mésolithique au Néolithique, l'évolution du paysage végétal dans le bassin de de la Loire océanique et moyenne. In: *Approche archéologique de l'environnement et de l'aménagement du territoire lignien*. Notes: Acte de Colloque. Fédération Archéologique du Loiret, 117-123.

Gauthier, G., Vannière, B., & Martineau R. (2005). Histoire des feux et pratiques agraires du Néolithique à l'âge du Fer en région Centre : implications territoriales démographiques et environnementales. *Gallia préhistoire,* 47:167-186.
