# Supplementary material for: Signature of mid‐Pleistocene lineages in the European silver fir (Abies alba Mill.) at its geographic distribution margin
Source: Ecol Evol. 2021 Jul 21;11(16):10984–99. doi: 10.1002/ece3.7886 (PMC8366861; doi:10.1002/ece3.7886)
Supplement: Supplementary file 1 — Appendix S1‐S5 [file ECE3-11-10984-s001.zip › Appendix_S3_Demography_PhylogeoPyr_Aalba_07052021.doc]

***SUPPORTING INFORMATION***

**Signature of mid-Pleistocene lineages in the European silver fir (*Abies alba* Mill.) at its geographic distribution margin.**

**Appendix S3:** details of the demographic analyses

**Figure S3.1:** Evaluation of the simulated data sets to test for the scenarios of divergence: Principal component analysis. Results obtained for the first set of four populations (Borc, Stla, Lesp, Vent).

**Figure S3.2:** Posterior probability of scenarios: logistic regression. Results obtained for the first set of four populations (Borc, Stla, Lesp, Vent).

**Figure S3.3:** Model checking for the best scenario of divergence. Results obtained for the first set of four populations (Borc, Stla, Lesp, Vent).

**Figure S3.4:** Comparison of the modes of the ratio between current and ancestral population sizes (N_current_/N_past_) in the four genetic clusters (Western Pyrenees, Eastern Pyrenees, Pyrenees admixed zone, Alps). Results obtained for the first set of four populations (Borc, Stla, Lesp, Vent).

**Figure S3.5:** Posterior probability of scenarios: logistic regression. Results obtained for the second set of four populations (Issa, Sigu, Bele, Punt).

**Figure S3.6:** Inference of the time parameters in generations for scenarios S1, S2, S3, S4. Results are obtained for the two sets of four populations (Borc, Stla, Lesp, Vent (Ana1) and Issa, Sigu, Bele, Punt (Ana2) representative of the four genetic clusters: Eastern genetic cluster, Admix genetic cluster, Western genetic cluster and Southwestern Alps genetic clusters respectively. For each analysis, the median and the limits of the 2.5% and 97.5% coverage are reported.

**Figure S3.7:** Inference of the demographic size parameters in generations for scenarios S1, S2, S3, S4. Results are obtained for the two sets of four populations (Borc, Stla, Lesp, Vent (Ana1) and Issa, Sigu, Bele, Punt (Ana2) representative of the four genetic clusters: Eastern genetic cluster, Admix genetic cluster, Western genetic cluster and Southwestern Alps genetic clusters respectively. For each analysis, the median and the limits of the 2.5% and 97.5% coverage are reported.

**Table S3.1:** Prior distribution of the mutation parameters used in DIYABC. Results obtained for the first set of four populations (Borc, Stla, Lesp, Vent).

**Table S3.2:** Summary statistics used for the simulations of the demographic scenario and used for the goodness-of-fit of the best scenario. Results obtained for the first set of four populations (Borc, Stla, Lesp, Vent).

**Table S3.3:** Inference of the demographic size parameters for scenarios S1, S2, S3, S4. Results obtained for the first set of four populations (Borc, Stla, Lesp, Vent).

**Table S3.4:** Inference of the time parameters in generations for scenarios S1, S2, S3, S4. Results obtained for the first set of four populations (Borc, Stla, Lesp, Vent).

**Table S3.5:** Inference of the admixture rate parameters ra and rb parameters for scenarios S1, S2, S3, S4. Results obtained for the first set of four populations (Borc, Stla, Lesp, Vent).

**Table S3.6:** Inference of the mutation rate parameters for scenarios S1, S2, S3, S4. Results obtained for the first set of four populations (Borc, Stla, Lesp, Vent).

**Table S3.7**: Model checking for the best scenario of divergence (S2), test of rank. Results obtained for the first set of four populations (Borc, Stla, Lesp, Vent).

**Table S3.8**: Precision of parameter estimations for the best scenario of divergence (S2). Results obtained for the first set of four populations (Borc, Stla, Lesp, Vent).

**Table S3.9**: Inference of the demographic size parameters for scenario S1 S2, S3, S4. Results obtained for the second set of four populations (Issa, Sigu, Bele, Punt).

**Table S3.10:** Inference of the time parameters in generations for scenario S1 S2, S3, S4. Results obtained for the second set of four populations (Issa, Sigu, Bele, Punt).

**Table S3.11:** Inference of the admixture rate parameters ra and rb parameters for scenario S1 S2, S3, S4. Results obtained for the second set of four populations (Issa, Sigu, Bele, Punt).

**Table S3.12:** Inference of the mutation rate parameters for scenario S1 S2, S3, S4. Results obtained for the second set of four populations (Issa, Sigu, Bele, Punt).

**Figure S3.1:** Evaluation of the simulated data sets to test for the scenarios of divergence: Principal component analysis. Results obtained for the first set of four populations (Borc, Stla, Lesp, Vent).

The purpose of this evaluation is to check that at least one combination of scenarios and priors can produce simulated data sets that are close enough to the observed data set. A principal component analysis (PCA) is performed in the space of summary statistics on, at most, 100,000 simulated data set and the observed data are added on each plane (large yellow dot) of the PCA in order to evaluate how the latter is surrounded by simulated data sets (4 colors for each of the 4 scenarios of divergence tested).

**Figure S3.2:** Posterior probability of scenarios of divergence: logistic regression. a.) The four scenarios are compared b.) Scenarios 1 and 2 are compared, c.) Scenarios 2 and 3 are compared d.) Scenarios 2 and 4 are compared. Results obtained for the first set of four populations (Borc, Stla, Lesp, Vent).

A polychotomic weighted logistic regression is performed on the first 10% data sets with the proportion of the scenario as the dependent variable and the differences between observed and simulated data set summary statistics as the independent variables. The intercept of the regression (corresponding to an identity between simulated and observed summary statistics) is taken as the point estimate. (Cornuet et al., 2008). Four scenarios are compared in a.) and two scenario are compared in b.), c.), d.).

**a.**

**b.**

**c.**

**d.**

Cornuet, J. M., Santos, F., Beaumont, M. A., Robert, C. P., Marin, J. M., Balding, D. J., … Estoup, A. (2008). Inferring population history with DIY ABC: A user-friendly approach to approximate Bayesian computation. *Bioinformatics*, 24(23), 2713–2719

**Figure S3.3:** Model-checking evaluation. Results obtained for the first set of four populations (Borc, Stla, Lesp, Vent).

The adequacy, *i.e.* the ability of a given scenario to produce data sets similar to the real data set, of best scenario S2 was tested using the model checking option of DIYABC.

Large yellow dots represent how well the observed data fit the posterior predictive distributions (larger red dots). In (a), the summary statistics are those already used during the inference steps (model discrimination and estimation of parameters). Since there is a risk of over-estimating the quality of the fit by using the same statistics twice, we also performed the model checking using summary statistics that did not correspond to the summary statistics used for previous inferential steps (b). For each summary statistics, the discrepancy between simulated and observed data was then assessed by ranking the observed value among the values obtained with the simulated data sets. This provided an estimation of the *P*-value for the considered summary statistics (see Table S3.7).

Precision of parameter estimation was assessed by computing the relative median of the absolute error on 300 pseudo-observed data sets (that is, data sets simulated with known parameters) simulated with the best scenario. Relative median of the absolute error is the 50% quantile (over the 300 pseudo-observed data sets) of the absolute value of the difference between the median value of the posterior distribution sample (in each data set) and the true value, divided by the true value.

a.

b.

**Figure S3.4:** Comparison of the modes of the ratio between current and ancestral population sizes (N_current_/N_past_) in the four genetic clusters (Pyrenees (east, west and admixed clusters), Western Alps). Results obtained for the first set of four populations (Borc, Stla, Lesp, Vent).

**Figure S3.5:** Posterior probability of scenarios: logistic regression. Results obtained for the second set of four populations (Issa, Bele, Sigu, Punt).

**Figure S3.6:** Inference of the time parameters in generations for scenarios S1, S2, S3, S4. Results are obtained for the two sets of four populations (Borc, Stla, Lesp, Vent (Ana1) and Issa, Sigu, Bele, Punt (Ana2) representative of the four genetic clusters: Eastern genetic cluster, Admix genetic cluster, Western genetic cluster and Southwestern Alps genetic clusters respectively. For each analysis, the median and the limits of the 2.5% and 97.5% coverage are reported.

**Figure S3.7:** Inference of the demographic size parameters in generations for scenarios S1, S2, S3, S4. Results are obtained for the two sets of four populations (Borc, Stla, Lesp, Vent (Ana1) and Issa, Sigu, Bele, Punt (Ana2) representative of the four genetic clusters: Eastern genetic cluster, Admix genetic cluster, Western genetic cluster and Southwestern Alps genetic clusters respectively. For each analysis, the median and the limits of the 2.5% and 97.5% coverage are reported.

**Table S3.1:** Prior distribution of parameters used in DIYABC. Results obtained for the first set of four populations (Borc, Stla, Lesp, Vent).

| **Parameter** | **Distribution** | **Min-Max** | **Mean** | **other parameter** | **Justification of the choice** |
| --- | --- | --- | --- | --- | --- |
| **nuSSR mutation parameters** | | | | | |
| Mean mutation rate (*µNu_SSR_)* | Log uniform | 1.10^-4^-1.10^-3^ |  |  | See text bellow |
| Individual mutation rate | gamma |  | *µNu_SSR_* | Shape =2 | Default value |
| Mean coefficient P | uniform | 0.1-1 |  |  | Default value |
| Individual locus coefficient P | Gamma |  | Mean coefficient P | Shape =2 | Default value |
| **cpSSR mutation parameters** | | | | | |
| Mean mutation rate (*µCp_SSR_)* | Log uniform | 1.10^-8^-1.10^-3^ |  |  | See text bellow |
| Individual mutation rate | gamma |  | *µCp_SSR_* | Shape =2 | Default value |
| Mean coefficient P | uniform | 0.1-1 |  |  | Default value |
| Individual locus coefficient P | Gamma |  | Mean coefficient P | Shape =2 | Default value |
| Individual locus coefficient k_C/T (Gamma distribution around mean) | Gamma |  | Mean coefficient k_C/T | Shape =2 | Default value |
| **Time scale in generations** | normal |  | 25000 | Standard deviation = 12500 | See text bellow |
| **Effective population size** | normal |  | 5000 | Standard deviation = 2500 | See text bellow |
| **Admixture parameter** | normal |  | 0.5 | 0.25 | See text bellow |

**Explanation of the choice of the priors:**

In the absence of knowledge on the mutation rate of microsatellite markers in fir, the prior distributions were chosen according to previous studies:

for the nuSSRs we choose a mean mutation rate (*µNu_SSR_*) sampled from a log uniform prior distribution between 1.10^-4^-1.10^-3^ because of the results obtained in Arabidopsis by Marriage et al., (2009) showing that the average mutation rate per generation was 8.87 × 10−4 (SE 2.57 × 10−4). We allowed variation between loci by drawing an individual mutation rate within a gamma distribution of mean equal to *mu_mean* and a shape of two (default values).

for the cpSSRs we choose a mean mutation rate (*µCp_SSR_*) sampled from a log uniform prior distribution between 1.10^-3^-1.10^-8^ which encompassed the results obtained by Provan et al., (1999) showing the mutation rate at mononucleide repeat cpSSR was between 3.2.10-5 and 7.9.10-5. we chose to increase the higher limit value to 10-3 to include the values used in a recent paper on Spanish fir (Cobo-Simon et al., 2020). We also decreased the interval to 1.10-8 due to the presence in our study of a minisatellites (repetition of 18 bp), this value is ten time higher than the mutation rate obtained in cp gene sequences by Wolfe et al., 1987 that constitute an upper limit (1.-10-9 – 3.10-9). As for nuSSR we allowed variation between loci by drawing an individual mutation rate within a gamma distribution of mean equal to *mu_mean* and a shape of two (default values).

For Time, we built the prior distribution based on the recent knowledge on time of divergence between closely related species of *abies alba* and *abies nordmaniana* which diverge around 25,000 generations ago (Hrivnak et al., 2017). To consider, the uncertainties surrounding this estimate we have chosen to consider a wider distribution with a mean centred at 25,000 (equivalent to 1 million years if we consider a flowering age time of 50 years in average) and large standard deviation equal to 12,500 generations but constrained by a minimum of 10 and a maximum of 50,000 generations.

For Ne, the effective population size we begun by drawing values from a normal distribution of mean 50,000 and standard deviation, sd=25,000) that encompassed recent estimates in *abies alba* sampled in Bulgaria and its related species *A. nordmanniana* in eastern Europe (Hrivnak et al., 2017). However, after using the option “evaluate scenario-prior combination” we showed that half of the nuSSR summary statistics were far to the observed summary statistics: After performing a PCA, when the observed data set is added in the space of summary statistics calculated from 1 million simulated data sets, the observed data set is off-center revealing misspecification of the prior distribution. Based on another recent study (Cobo-Simon et al., 2020), we changed the mean value of the prior distribution Ne (mean = 5000 and standard deviation = 2500) to encompass the values of the ancestral population of the Spanish fir (Ne=7000) before it underwent a contraction during the Pleistocene. These values have been retained and the PCA is given in figure S3.1.

For the admixture parameter, in absence of knowledge, we used a normal distribution of mean equal to 0.5 with a large standard deviation allowing to explore a large interval of values.

Cobo-Simón I., Méndez-Cea B., Jump A., Seco J., Gallego FLinares J., Linares J.C., 2020. Understanding genetic diversity of relict forests. Linking long-term isolation legacies and current habitat fragmentation in Abies pinsapo Boiss. Forest Ecology and Management, 117947, 461.

Hrivnák M, Paule L., Krajmerová D., Kulaç Ş., Şevik H., Turna İ., Tvauri I., Gömöry D. 2017. Genetic variation in Tertiary relics: The case of eastern-Mediterranean Abies (Pinaceae.Ecol. Evol., 7 (2017), pp. 10018-10030.

Marriage, T., Hudman, S., Mort, M. et al. Direct estimation of the mutation rate at dinucleotide microsatellite loci in Arabidopsis thaliana (Brassicaceae). Heredity 103, 310–317 2009

Provan J, Soranzo N, Wilson NJ, Goldstein DB, Powell WA (1999b) Low mutation rate for chloroplast microsatellites. Genetics 153:943–947

Wolfe, K. H., W. H. Li and P. M. Sharp, 1987 Rates of nucleotide substitution vary greatly among plant mitochondrial, chloroplast and nuclear DNA. Proc. Natl. Acad. Sci. USA 84: 9054–9058.

**Table S3.2***:* Summary statistics used for the simulations of the demographic scenarios and for the goodness-of-fit tests of the best scenario. Results obtained for the first set of four populations (Borc, Stla, Lesp, Vent).

| *Simulations of the demographic scenarios* | | |
| --- | --- | --- |
| Single-population summary statistics | | |
|  | mean number of alleles | nuSSR and cpSSR |
|  | mean genic diversity (Nei, 1987) | cpSSR |
|  | mean allele size variance | nuSSR and cpSSR |
|  | mean Garza-Williamson's M (Garza et al. 2001) | cpSSR |
| Two-populations summary statistics | | |
|  | mean number of alleles between two samples | cpSSR |
|  | mean allele size variance across loci between two samples | nuSSR and cpSSR |
|  | F_ST_ between two samples (Weir and Cockerham, 1984) | nuSSR and cpSSR |
|  | (δμ)^2^ distance between two samples (Goldstein et al., 1995) | nuSSR and cpSSR |
| *Goodness-of-fit of the best scenario* | | |
| Single-population summary statistics | | |
|  | Mean genic diversity | nuSSR and cpSSR |
|  | Mean Garza Williamson's M | nuSSR |
| Two-populations summary statistics | | |
|  | Mean number of alleles | nuSSR |
|  | Mean genic diversity | nuSSR and cpSSR |

Garza JC and E Williamson, 2001. Detection of reduction in population size using data from microsatellite DNA. Mol. Ecol. 10,305-318.

Goldstein DB, Linares AR, Cavalli-Sforza LL, and Feldman MW, 1995. An evaluation of genetic distances for use with microsatellite loci. Genetics 139, 463-471

Nei M., 1987. Molecular Evolutionary Genetics. Columbia University Press, New York, 512 pp.

Weir BS and CC Cockerham, 1984. Estimating F-statistics for the analysis of population structure. Evolution 38: 1358-1370.

**Table S3.3:** Inference of the demographic size parameters for scenarios S1, S2, S3, S4. Results obtained for the first set of four populations (Borc, Stla, Lesp, Vent).

| **Parameter** | **Genetic cluster** | **Scenario** | **median** | **2.5% coverage** | **97.5% coverage** |  |
| --- | --- | --- | --- | --- | --- | --- |
| **Current demographic size N** | Western Alps cluster | S1 | 7880 | 5690 | 9570 |  |
|  |  | S2 | 8030 | 5860 | 9630 |  |
|  |  | S3 | 8030 | 5820 | 9630 |  |
|  |  | S4 | 8060 | 5830 | 9600 |  |
|  | Eastern Pyrenees cluster | S1 | 7640 | 5310 | 9530 |  |
|  |  | S2 | 7940 | 5730 | 9580 |  |
|  |  | S3 | 7150 | 4710 | 9380 |  |
|  |  | S4 | 7590 | 5310 | 9500 |  |
|  | Western Pyrenees cluster | S1 | 4470 | 2380 | 7890 |  |
|  |  | S2 | 4310 | 2260 | 7740 |  |
|  |  | S3 | 3980 | 2030 | 7490 |  |
|  |  | S4 | 4330 | 2210 | 7720 |  |
|  | Admixed cluster | S1 | 7570 | 5300 | 9450 |  |
|  |  | S2 | 8000 | 5880 | 9520 |  |
|  |  | S3 | 8150 | 6160 | 9590 |  |
|  |  | S4 | 7620 | 5300 | 9430 |  |
| **Ancestral demographic size N** |  | S1 | 3660 | 465 | 8560 |  |
|  |  | S2 | 4670 | 747 | 9010 |  |
|  |  | S3 | 4830 | 724 | 9040 |  |
|  |  | S4 | 4420 | 601 | 8930 |  |
| **Demographic size before resizing** | Western Alps cluster | S1 | 5150 | 898 | 9280 |  |
|  |  | S2 | 5730 | 1220 | 9390 |  |
|  |  | S3 | 5580 | 1080 | 9340 |  |
|  |  | S4 | 6090 | 1260 | 9430 |  |
|  | Eastern Pyrenees cluster | S1 | 5280 | 899 | 9280 |  |
|  |  | S2 | 4550 | 663 | 9020 |  |
|  |  | S3 | 4770 | 803 | 9090 |  |
|  |  | S4 | 5200 | 898 | 9240 |  |
|  | Western Pyrenees cluster | S1 | 4720 | 811 | 9140 |  |
|  |  | S2 | 4550 | 693 | 9070 |  |
|  |  | S3 | 5140 | 941 | 9240 |  |
|  |  | S4 | 3400 | 458 | 8470 |  |
|  | Admixed cluster | S1 | 4980 | 933 | 9170 |  |
|  |  | S2 | 4790 | 753 | 9120 |  |
|  |  | S3 | 5290 | 891 | 9170 |  |
|  |  | S4 | 5310 | 1040 | 9230 |  |

**Table S3.4:** Inference of the time parameters in generations for scenarios S1, S2, S3, S4. Results obtained for the first set of four populations (Borc, Stla, Lesp, Vent).

| **Parameter** | **Genetic cluster** | **Scenario** | **median** | **2.5% coverage** | **97.5% coverage** |
| --- | --- | --- | --- | --- | --- |
| **Divergence time, T1** | Alps/Eastern Pyrenees/Western Pyrenees | S1 | 24000 | 11600 | 45700 |
|  | Alps/Western Pyrenees | S2 | 27000 | 13600 | 46900 |
|  | Alps/Eastern Pyrenees | S3 | 29300 | 15700 | 47500 |
|  | Alps/Western | S4 | 31200 | 17900 | 47900 |
| **Divergence time, T2** | Western Pyrenees/Eastern Pyrenees | S2 | 22900 | 12900 | 38100 |
|  | Alps/Eastern Pyrenees | S3 | 25000 | 14600 | 39200 |
|  | Alps/Western Pyrenees | S4 | 24400 | 14200 | 39300 |
| **Admixture time, T3** | Eastern Pyrenees + Western Pyrenees | S1 | 23000 | 12700 | 37900 |
|  | Eastern Pyrenees + Western Pyrenees | S2 | 20400 | 11400 | 32500 |
|  | Eastern Pyrenees + Western Pyrenees | S3 | 22200 | 12700 | 34100 |
|  | Eastern Pyrenees + Western Pyrenees | S4 | 21500 | 12300 | 33700 |
| **Resizing time** | Western Alps | S1 | 15600 | 2010 | 30600 |
|  |  | S2 | 13700 | 1730 | 26900 |
|  |  | S3 | 17700 | 2350 | 31500 |
|  |  | S4 | 16700 | 2520 | 30500 |
|  | Eastern Pyrenees | S1 | 15100 | 2080 | 30300 |
|  |  | S2 | 13400 | 1750 | 26600 |
|  |  | S3 | 14100 | 1850 | 27700 |
|  |  | S4 | 18100 | 2530 | 31800 |
|  | Western Pyrenees | S1 | 17300 | 2270 | 32400 |
|  |  | S2 | 13900 | 1600 | 26900 |
|  |  | S3 | 15100 | 1960 | 28700 |
|  |  | S4 | 14800 | 1980 | 28200 |
|  | Admixed | S1 | 18100 | 2500 | 33500 |
|  |  | S2 | 13900 | 1820 | 27400 |
|  |  | S3 | 13700 | 1670 | 27300 |
|  |  | S4 | 12800 | 1590 | 25900 |

**Table S3.5:** Inference of the admixture rate parameters ra and rb for scenarios S1, S2, S3, S4. Results obtained for the first set of four populations (Borc, Stla, Lesp, Vent).

| **Parameter** | **Genetic cluster** | **Scenario** | **median** | **2.5% coverage** | **97.5% coverage** |
| --- | --- | --- | --- | --- | --- |
| **ra** (Pyrenees contribution) | Eastern Pyrenees + Alps | S3 | 0.49 | 0.0802 | 0.915 |
|  | Western Pyrenees + Alps | S4 | 0.651 | 0.139 | 0.953 |
| **rb** (Western Pyrenees contribution) | Eastern Pyrenees + Western Pyrenees | S1 | 0.505 | 0.0818 | 0.921 |
|  | Eastern Pyrenees + Western Pyrenees | S2 | 0.52 | 0.0904 | 0.923 |
|  | Eastern Pyrenees + Western Pyrenees | S3 | 0.55 | 0.097 | 0.93 |
|  | Eastern Pyrenees + Western Pyrenees | S4 | 0.48 | 0.0787 | 0.916 |

**Table S3.6:** Inference of the mutation rate parameters for scenarios S1, S2, S3, S4. Results obtained for the first set of four populations (Borc, Stla, Lesp, Vent).

| **Parameter** | **marker** | **Scenario** | **median** | **2.5% coverage** | **97.5% coverage** |
| --- | --- | --- | --- | --- | --- |
| **mutation rate µ** | nSSR | S1 | 0.000147 | 0.000105 | 0.000326 |
|  |  | S2 | 0.000164 | 0.000109 | 0.000383 |
|  |  | S3 | 0.000172 | 0.00011 | 0.000387 |
|  |  | S4 | 0.000169 | 0.000109 | 0.000395 |
|  | cpSSR | S1 | 0.000319 | 0.000109 | 0.000878 |
|  |  | S2 | 0.000322 | 0.000111 | 0.000881 |
|  |  | S3 | 0.000345 | 0.000123 | 0.000895 |
|  |  | S4 | 0.000282 | 0.000095 | 0.000857 |
| **mutation rate's parameter** | nSSR (geometric distribution) | S1 | 0.262 | 0.115 | 0.798 |
|  |  | S2 | 0.324 | 0.122 | 0.855 |
|  |  | S3 | 0.269 | 0.115 | 0.78 |
|  |  | S4 | 0.267 | 0.116 | 0.788 |
|  | cpSSR (geometric distribution) | S1 | 0.413 | 0.133 | 0.952 |
|  |  | S2 | 0.411 | 0.132 | 0.95 |
|  |  | S3 | 0.432 | 0.136 | 0.962 |
|  |  | S4 | 0.42 | 0.134 | 0.954 |

**Table S3.7**: Model checking for the best scenario of divergence (S2), test of rank. Results obtained for the first set of four populations (Borc, Stla, Lesp, Vent).

For each summary statistics, the discrepancy between simulated and observed data was assessed by ranking the observed value among the values obtained with the simulated data sets. This provided an estimation of the *P*-value for the considered summary statistics. The Probability (summary statistics _simulated_ < summary statistics _observed_) is given for each summary statistics. To avoid the risk of over-estimating the quality of the fit, we used different summary statistics than the ones used during the inference steps (model discrimination and posterior estimation of parameters). The simulated summary statistics were obtained from 1000 new data sets that were simulated from the posterior distribution of parameters.

The code used for the summary statistics is composed of three parts: the summary statistic (see above for the definitions), the type of marker (1,2 for nSSR, cpSSR, respectively), the population cluster (1,2,3,4) for Western Pyrenees, Eastern Pyrenees, Western Alps, Admixed W-E Pyrenees. *HET*: mean genic diversity within population, *MGW*: Mean Garza Williamson's M, *N2P*: Mean number of alleles between pairs of populations *H2P:* mean genic diversity between pairs of populations.

**Table S3.8**: Precision of parameter estimations for the best scenario of divergence (S2). Results obtained for the first set of four populations (Borc, Stla, Lesp, Vent).

RMAE is the relative median of the absolute error. RMAE taking the median of the posterior distribution as point estimates was computed on 1000 pseudo-observed data sets simulated using the best scenario

**Table S3.9:** Inference of the demographic size parameters for scenario S1 S2, S3, S4. Results obtained for the second set of four populations (Issa, Sigu, Bele, Punt).

| **Parameter** | **Genetic cluster** | **Scenario** | **median** | **2.5% coverage** | **97.5% coverage** |
| --- | --- | --- | --- | --- | --- |
| **Current demographic size N** | Western Alps cluster | S1 | 8440 | 6540 | 9700 |
|  |  | S2 | 8520 | 6690 | 9720 |
|  |  | S3 | 8590 | 6770 | 9760 |
|  |  | S4 | 8500 | 6530 | 9740 |
|  | Eastern Pyrenees cluster | S1 | 8160 | 6100 | 9610 |
|  |  | S2 | 8010 | 5940 | 9600 |
|  |  | S3 | 8160 | 6100 | 9600 |
|  |  | S4 | 7920 | 5720 | 9490 |
|  | Western Pyrenees cluster | S1 | 6180 | 3680 | 8970 |
|  |  | S2 | 5860 | 3400 | 8920 |
|  |  | S3 | 6140 | 3620 | 9000 |
|  |  | S4 | 5970 | 3520 | 8900 |
|  | Admixed cluster | S1 | 7860 | 5590 | 9480 |
|  |  | S2 | 7640 | 5450 | 9470 |
|  |  | S3 | 7780 | 5570 | 9490 |
|  |  | S4 | 7830 | 5730 | 9480 |
| **Ancestral demographic size N** |  | S1 | 3550 | 455 | 8500 |
|  |  | S2 | 4430 | 652 | 8920 |
|  |  | S3 | 4500 | 639 | 9050 |
|  |  | S4 | 4560 | 675 | 8990 |
| **Demographic size before Resizing** | Western Alps cluster | S1 | 5540 | 980 | 9330 |
|  |  | S2 | 5080 | 923 | 9220 |
|  |  | S3 | 4710 | 764 | 9070 |
|  |  | S4 | 6170 | 1290 | 9490 |
|  | Eastern Pyrenees cluster | S1 | 4650 | 712 | 9130 |
|  |  | S2 | 4650 | 701 | 9100 |
|  |  | S3 | 4500 | 660 | 8950 |
|  |  | S4 | 4880 | 800 | 9170 |
|  | Western Pyrenees cluster | S1 | 4550 | 756 | 9020 |
|  |  | S2 | 5500 | 951 | 9300 |
|  |  | S3 | 5000 | 887 | 9260 |
|  |  | S4 | 4120 | 562 | 8940 |
|  | Admixed cluster | S1 | 5710 | 1130 | 9360 |
|  |  | S2 | 5380 | 904 | 9210 |
|  |  | S3 | 5190 | 892 | 9220 |
|  |  | S4 | 5480 | 997 | 9350 |
|  |  |  |  |  |  |

**Table S3.10:** Inference of the time parameters in generations for scenario S1 S2, S3, S4. Results obtained for the second set of four populations (Issa, Sigu, Bele, Punt).

| **Parameter** | **Genetic cluster** | **Scenario** | **median** | **2.5% coverage** | **97.5% coverage** |
| --- | --- | --- | --- | --- | --- |
| **Divergente time, T1** | Alps/Eastern Pyrenees/Western Pyrenees | S1 | 24600 | 12000 | 45500 |
|  | Alps/Western Pyrenees | S2 | 26400 | 13600 | 46700 |
|  | Alps/Eastern Pyrenees | S3 | 25000 | 11900 | 46000 |
|  | Alps/Western | S4 | 25300 | 12600 | 46600 |
| **Divergente time, T2** | Western Pyrenees/Eastern Pyrenees | S2 | 21000 | 11700 | 36000 |
|  | Alps/Eastern Pyrenees | S3 | 21800 | 12000 | 36900 |
|  | Alps/Western Pyrenees | S4 | 21700 | 12000 | 36700 |
| **Admixture time, T3** | Eastern Pyrenees + Western Pyrenees | S1 | 21500 | 11500 | 36800 |
|  | Eastern Pyrenees + Western Pyrenees | S2 | 19500 | 11000 | 31400 |
|  | Eastern Pyrenees + Western Pyrenees | S3 | 18300 | 9790 | 30100 |
|  | Eastern Pyrenees + Western Pyrenees | S4 | 19800 | 11000 | 32000 |
| **Resizing Time** | Western Alps | S1 | 18300 | 2570 | 33400 |
|  |  | S2 | 16300 | 2560 | 30400 |
|  |  | S3 | 14600 | 2050 | 28100 |
|  |  | S4 | 13100 | 1710 | 26100 |
|  | Eastern Pyrenees | S1 | 17400 | 2420 | 32700 |
|  |  | S2 | 13900 | 1910 | 26800 |
|  |  | S3 | 14500 | 2020 | 27800 |
|  |  | S4 | 14800 | 1910 | 28600 |
|  | Western Pyrenees | S1 | 14100 | 1910 | 29000 |
|  |  | S2 | 12500 | 1580 | 25600 |
|  |  | S3 | 12100 | 1450 | 25100 |
|  |  | S4 | 11900 | 1480 | 24900 |
|  | Admixed | S1 | 12400 | 1590 | 27100 |
|  |  | S2 | 15000 | 1910 | 28800 |
|  |  | S3 | 14400 | 1760 | 28000 |
|  |  | S4 | 13000 | 1620 | 26000 |

**Table S3.11:** Inference of the admixture rate parameters ra and rb parameters for scenario S1 S2, S3, S4. Results obtained for the second set of four populations (Issa, Sigu, Bele, Punt).

| **Parameter** | **Genetic cluster** | **Scenario** | **median** | **2.5% coverage** | **97.5% coverage** |
| --- | --- | --- | --- | --- | --- |
| **ra**  (Pyrenees contribution) | Eastern Pyrenees + Alps | S3 | 0.566 | 0.102 | 0.934 |
|  | Western Pyrenees + Alps | S4 | 0.565 | 0.103 | 0.940 |
| **rb**  (Western Pyrenees contribution) | Eastern Pyrenees + Western Pyrenees | S1 | 0.554 | 0.098 | 0.931 |
|  | Eastern Pyrenees + Western Pyrenees | S2 | 0.566 | 0.100 | 0.937 |
|  | Eastern Pyrenees + Western Pyrenees | S3 | 0.569 | 0.109 | 0.935 |
|  | Eastern Pyrenees + Western Pyrenees | S4 | 0.461 | 0.075 | 0.912 |

**Table S3.12:** Inference of the mutation rate parameters for scenario S1 S2, S3, S4. Results obtained for the second set of four populations (Issa, Sigu, Bele, Punt).

| **Parameter** | **marker** | **Scenario** | **median** | **2.5% coverage** | **97.5% coverage** |
| --- | --- | --- | --- | --- | --- |
| **mutation rate µ** | nSSR | S1 | 0.000147 | 0.000105 | 0.000326 |
|  |  | S2 | 0.000164 | 0.000109 | 0.000383 |
|  |  | S3 | 0.000172 | 0.00011 | 0.000387 |
|  |  | S4 | 0.000169 | 0.000109 | 0.000395 |
|  | cpSSR | S1 | 0.000319 | 0.000109 | 0.000878 |
|  |  | S2 | 0.000322 | 0.000111 | 0.000881 |
|  |  | S3 | 0.000345 | 0.000123 | 0.000895 |
|  |  | S4 | 0.000282 | 0.000095 | 0.000857 |
| **mutation rate's parameter** | nSSR (geometric distribution) | S1 | 0.262 | 0.115 | 0.798 |
|  |  | S2 | 0.324 | 0.122 | 0.855 |
|  |  | S3 | 0.269 | 0.115 | 0.780 |
|  |  | S4 | 0.267 | 0.116 | 0.788 |
|  | cpSSR (geometric distribution) | S1 | 0.413 | 0.133 | 0.952 |
|  |  | S2 | 0.411 | 0.132 | 0.950 |
|  |  | S3 | 0.432 | 0.136 | 0.962 |
|  |  | S4 | 0.420 | 0.134 | 0.954 |
