# Supplementary material for: Signature of mid‐Pleistocene lineages in the European silver fir (Abies alba Mill.) at its geographic distribution margin
Source: Ecol Evol. 2021 Jul 21;11(16):10984–99. doi: 10.1002/ece3.7886 (PMC8366861; doi:10.1002/ece3.7886)
Supplement: Supplementary file 1 — Appendix S1‐S5 [file ECE3-11-10984-s001.zip › Appendix_S4_Correlations_PhylogeoPyr_Aalba_final.doc]

***SUPPORTING INFORMATION***

**Signature of mid-Pleistocene lineages in the European silver fir (*Abies alba* Mill.) at its geographic distribution margin.**

***Appendix S4: Correlations between genetic parameters and environmental variables***

**Table S4.1:** List and definition of the environmental variables used to test for correlations with genetic parameters.

**Table S4.2:** Environmental characteristics for the Pyrenees populations. See separate file “Appendix_S4_Correlations_TableS4.2_PhylogeoPyr_Aalba_final”

**Table S4.3:** Pearson correlations between genetic and environmental variables. See separate file “Appendix_S4_Correlations_TableS4.3_PhylogeoPyr_Aalba_final”

**Table S4.4:** Results of the forward stepwise variable selection procedure on generalized linear models. See separate file “Appendix_S4_Correlations_TableS4.4_PhylogeoPyr_Aalba_final”

**Figure S4.1:** Summary of the forward stepwise variable selection procedure.

**Table S4.1.** List and definition of the environmental variables used to test for correlations with genetic parameters

| **ENVIRONMENTAL VARIABLES** | **DEFINITION** | |
| --- | --- | --- |
| **bioclimatic variables (WorldClim 1.4, Hijmans et al., 2005)** | | |
| Bio 1 | Annual Mean Temperature | |
| Bio 2 | Mean Diurnal Range | |
| Bio 3 | Isothermality | |
| Bio 4 | Temperature Seasonality | |
| Bio 5 | Max Temperature of Warmest Month | |
| Bio 6 | Min Temperature of Coldest Month | |
| Bio 7 | Temperature Annual Range | |
| Bio 8 | Mean Temperature of Wettest Quarter | |
| Bio 9 | Mean Temperature of Driest Quarter | |
| Bio 10 | Mean Temperature of Warmest Quarter | |
| Bio 11 | Mean Temperature of Coldest Quarter | |
| Bio 12 | Annual Precipitation | |
| Bio 13 | Precipitation of Wettest Month | |
| Bio 14 | Precipitation of Driest Month | |
| Bio 15 | Precipitation Seasonality | |
| Bio 16 | Precipitation of Wettest Quarter | |
| Bio 17 | Precipitation of Driest Quarter | |
| Bio 18 | Precipitation of Warmest Quarter | |
| Bio19 | Precipitation of Coldest Quarter | |
| **Bioclimatic variables at four geological times** | | |
| Present | Present at 30 arc-seconds resolution | |
| MH | Mid Holocene, approx. 6,000 years before present, at 30 arc-seconds resolution | |
| LGM | Last Glacial Maximum (LGM, approx. 22,000 years BP) 2.5 arc-minutes resolution | |
| LIG | Last inter-glacial (LIG, approx. between 120,000 – 140,000 years BP), at 30 arc-seconds resolution | |
| **Other environmental variables:** | |  |
| Long | Longitude | |
| Lat | Latitude | |
| Slope | Slope | |
| Elevation | Elevation | |

Environmental characteristics were obtained for each population: slope, altitude, latitude, longitude. In addition, nineteen nine bioclimate variables were extracted from the WorldClim database (Hijmans et al., 2005). For the present climate (http://worldclim.org/version1, (Fick & Hijmans, 2017) we downloaded the 19 standard WorldClim bioclimatic variables average for the years 1970-2000 considering the 2.5 minutes resolution. For the past climate (http://worldclim.org/version1, Hijmans et al., 2005), predicted distributions during the Last Glacial Maximum (LGM; approx. 22,000 years ago) and the Mid-Holocene (MH: about 6000 years ago) of the 19 bioclimatic variables were obtained from the Community Climate System Model 4 (CCSM4; http://www.worldclim.org/paleo-climate) at 2.5 arc-minutes resolution. Predicted distribution during the Last inter-glacial (LIG: 120 000 – 140 000 years BP) of the bioclimatic variables were obtained from (Otto-Bliesner et al., 2006) at 30 arc-seconds resolution. Climate data from geospatial databases in raster format (GeoTiff) were combined into layers and converted to a raster stack using the ‘rgdal’ (Bivand, Keitt, & Rowlingson, 2017) and ‘raster’ (Hijmans, 2017) packages in R v3.2.3 (R Core Team, 2015). The raster stack was cropped to the relevant area (western border of Pyrenees to the eastern border of Alps) using the rasterVis library. Using the sp package, for each population, a spatial polygon was drawn connecting several corners-points, allowing to define a sampling area including four grid-points. The final step involves the extraction of the mean bioclimatic variables from the points encircled by the polygon.

Bivand, R., Keitt, T., Rowlingson, B., Pebesma, E., Sumner, M., Hijmans, R. J., & Rouault, E. (2017). rgdal: Bindings for the geospatial data abstraction library (Version R package version 1.2‐7). Retrieved from https://cran.r-project.org/package=rgdal.

Fick, S.E. and Hijmans, R.J. (2017), WorldClim 2: new 1‐km spatial resolution climate surfaces for global land areas. Int. J. Climatol, 37: 4302-4315. https://doi.org/10.1002/joc.5086

Hijmans, R. J., Cameron, S. ., Parra, J. L., Jones, P. G., & Jarvis, A. (2005). Very high resolution interpolated climate surfaces for global land areas. International Journal of Climatology, 25, 1965–1978.

Hijmans, R. J., van Etten, J., Cheng, J., Mattiuzzi, M., Sumner, M., Greenberg, J. A., … Shortridge, A. (2016). raster: Geographic data analysis and modeling (Version R package version 2.5‐8). Retrieved from http://cran.r-project.org/package=raster

Otto-Bliesner B.L., Marshall S.J., Overpeck J.T., Miller G.H., Hu A., et al. Simulating Arctic climate warmth and icefield retreat in the last interglaciation. Science, 311 (2006), pp. 1751-1753

R Core Team, (2015) R: A language and environment for statistical computing. R Foundation for Statistical Computing, Vienna, Austria. URL https://www.R-project.org/.

RStudio Team (2016). RStudio: Integrated Development for R. RStudio, Inc., Boston, MA URL http://www.rstudio.com/.

**Figure S4.1:** Summary of the forward stepwise variable selection procedure.

Out of a total of 37 significant regressions involving bioclimatic variables, this mosaic plot shows the number of significant regressions as a function of the period of inference of the variable (present, mid-Holocene, Last Glacial Maximum, Last Inter-Glacial) and the type of bioclimatic variable (related to temperature or precipitation). The y-axis represents the number of significant regressions for nuSSR and cpSSR markers and the number of non-significant regressions (ns). The area of the tiles (also known as the bin size) is proportional to the number of observations within that category.

Time period

Present

MH

LGM

LIG

Climatic variable

R Core Team, (2015) R: A language and environment for statistical computing. R Foundation for Statistical Computing, Vienna, Austria. URL <https://www.R-project.org/>.

RStudio Team (2016). RStudio: Integrated Development for R. RStudio, Inc., Boston, MA URL <http://www.rstudio.com/>.
